# Local inhibition of microtubule dynamics by dynein is required for neuronal cargo distribution

**DOI:** 10.1038/ncomms15063

**Published:** 2017-04-13

**Authors:** Shaul Yogev, Celine I. Maeder, Roshni Cooper, Mark Horowitz, Adam G. Hendricks, Kang Shen

**Affiliations:** 1 Department of Biology, Howard Hughes Medical Institute, Stanford University, 385 Serra Mall, California 94305, USA; 2 Department of Electrical Engineering, Stanford University, 350 Serra Mall, California 94305, USA; 3 Department of Electrical Engineering and Computer Science, Stanford University, 353 Serra Mall, California 94305, USA; 4 Department of Bioengineering, McGill University, 817 Sherbrooke Street West, Montreal, Quebec, Canada H3A 0C3

## Abstract

Abnormal axonal transport is associated with neuronal disease. We identified a role for DHC-1, the *C. elegans* dynein heavy chain, in maintaining neuronal cargo distribution. Surprisingly, this does not involve dynein's role as a retrograde motor in cargo transport, hinging instead on its ability to inhibit microtubule (MT) dynamics. Neuronal MTs are highly static, yet the mechanisms and functional significance of this property are not well understood. In disease-mimicking *dhc-1* alleles, excessive MT growth and collapse occur at the dendrite tip, resulting in the formation of aberrant MT loops. These unstable MTs act as cargo traps, leading to ectopic accumulations of cargo and reduced availability of cargo at normal locations. Our data suggest that an anchored dynein pool interacts with plus-end-out MTs to stabilize MTs and allow efficient retrograde transport. These results identify functional significance for neuronal MT stability and suggest a mechanism for cellular dysfunction in dynein-linked disease.

## Abstract (web summary)

Microtubule dynamics are essential for axonal transport. In *C. elegans*, the authors show that dynein heavy chain regulates the spatial distribution of dendritic microtubules which ensures correct transport progression.

The neuronal microtubule (MT) cytoskeleton provides structural support and serves as tracks for cargo transport. The large size of neurons necessitates unique properties for their MTs, which is reflected by neuron-specific MT-associated proteins and post-translational modifications^1^,^2^,^3^,^4^. The importance of functional MTs to the nervous system is underscored by neurodegenerative conditions associated with mutations in cytoskeletal components^5^,^6^.

One defining property of a MT polymer is its dynamic instability. *In vitro*, MTs frequently undergo transition from growth to shrinkage (catastrophe) and vice versa (rescue). *In vivo*, MT dynamics are regulated by a large number of cofactors that can anchor minus ends, promote polymerization or capture the growing end^7^,^8^,^9^,^10^.

Neuronal MTs are significantly less dynamic than MTs in most cell types, and they are more resistant to depolymerization by cold or Nocodazole^11^,^12^. In the axon, cold or drug-resistant MT seeds extend labile fragments^11^,^13^,^14^,^15^. Axonal MT dynamics reflect the interplay between stabilizing MAPs and tubulin polyamination and destabilizing factors such as depolymerizing kinesins, severing enzymes and Stathmins^1^,^3^,^4^,^16^,^17^,^18^. We recently developed a method that allows examining how this interplay shapes steady-state polymer length, abundance and distribution^19^. MT dynamics in the dendrite are less well understood. MAP2 likely plays a key role, but the knockout mice only exhibit a moderate reduction in MT density, suggesting the existence of additional mechanisms^20^,^21^. Importantly, the *in vivo* consequences of a loss of neuronal MT stability have not been explored.

Cytoplasmic dynein is the main minus-end-directed motor in eukaryotic cells. A heavy chain (DHC) dimer provides MT binding and energy production, and its activity and cargo specificity are regulated by a host of co-factors^22^,^23^,^24^. Dynein's most established role in neurons is in retrograde axonal transport, and impaired function of the heavy chain or other subunits leads to cargo trafficking defects^25^,^26^,^27^,^28^,^29^.

Mutations in human DHC (DYNC1H1) or accessory proteins lead to several symptoms. In particular, an N-terminal region, which mediates dimerization and assembly of the complex, seems to be a hotspot for mutations that result in spinal muscular atrophy (SMA)^30^,^31^,^32^,^33^,^34^.

In addition to transporting cargoes, immobilized dynein can move MTs, either in neurons or in non-neuronal cells. Studies in cultured sympathetic neurons implicated this function in populating the growing axon with plus-end-out MTs and in resisting contractile forces^35^,^36^,^37^. In cultured fly neurons dynein moves minus-end-out MTs out of the developing axon^38^.

In non-neuronal cells, anchored dynein can also serve as a plus-end tether^8^,^39^,^40^,^41^,^42^. *In vitro*, growing MTs buckle or shrink upon reaching a barrier, but when the barrier is coated with dynein, the MT filament straightens under tension induced by the motor^43^,^44^. These results suggest that anchored dynein can tether and stabilize MTs by interacting with their tips. Recently, MT tethering by dynein was implicated in synapse stabilization in cultured cortical neurons^41^.

Here we identified a role for DHC-1, the *Caenorhabditis elegans* dynein heavy chain, in maintaining the correct distribution of neuronal cargo. In *dhc-1* mutants, cargo accumulates at the tip of the dendrite and is concomitantly reduced at normal locations. Surprisingly, defective minus-end-directed transport is not driving this phenotype. Our data suggest that DHC-1 regulates the spatial distribution of dendritic MTs and suppresses MT dynamics. The latter function is required locally, at the tip of the dendrite, where excessively dynamic MTs buckle, twist and loop in *dhc-1* mutants. These unstable MTs trap cargo, leading to the ectopic accumulations and reduced availability of cargo at endogenous sites. We propose that anchored dynein tethers MT plus-ends at the dendrite tip to ensure correct cargo distribution. A similar mechanism may underlie some of the cellular defects that occur during dynein-related disease, as cargo trapping by unstable MTs is observed in a SMA-mimicking *dhc-1* allele.

## Results

### *wy743* mutants display ectopic cargo accumulations

**Figure 1:**
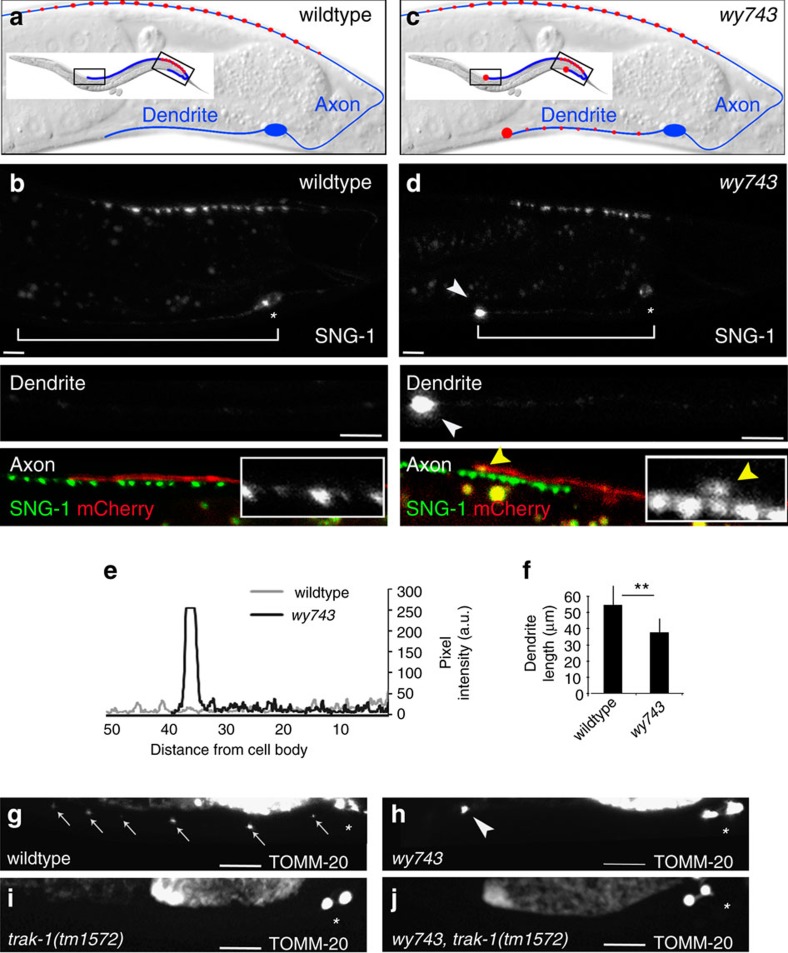
**Neuronal cargo accumulates at the tips of DA9 processes in** ***wy743*** **mutants**. (**a**) Schematic diagrams of the DA9 neuron. Inset shows the entire axon. (**b**) wt L4 larva (anterior is left and dorsal up in all figures) showing SVPs labelled by synaptogyrin/SNG-1::GFP. The cell body is marked by an asterisk. Lower panels show magnifications of the dendrite and the axon tip. Co-labelling with cytoplasmic mCherry (red) was used to identify the tip of the DA9 axon. Scale bar is 5 μm in all panels and figures. (**c**) Schematic diagram of the ectopic SVP accumulations in *wy743* dendrites. (**d**) synaptogirin/SNG-1::GFP accumulates at the tips of *wy743* processes. White arrowhead indicates the dendritic accumulation (top and middle panel), and yellow arrowheads point to SNG-1::GFP that mis-accumulates at the tip of the axon (lower panel). (**e**) Intensity plot of synaptogyrin/SNG-1::GFP fluorescence in wt (grey) and *wy743* (black) dendrites from **b** and **c**, showing the enrichment of SVPs at the distal tip. (**f**) Dendrite length is reduced in wy743 mutants. *n*=30 per genotype, *P*<0.01, *χ*^2^-test. (**g**,**h**) The mitochondrial marker TOMM-20::YFP is evenly distributed in wt dendrites (indicated by arrows in **g**) but accumulates at the tip in *wy743* mutants (arrowhead in **h**). (**i**) In *trak-1* mutants mitochondria do not enter the dendrite. (**j**) In *wy743; trak-1* double mutants, the mitochondria are confined to the cell body, suggesting that TOMM-20::YFP is correctly sorted.

The *C. elegans* cholinergic motor neuron DA9 sends out an anteriorly directed dendrite and a posterior axon that extends dorsally to join the dorsal nerve cord. A string of *en-passant* presynapses form in a restricted region of the axon (Fig. 1a). In wildtype (wt) L4 larvae, synaptic vesicle precursors (SVPs), labelled by synaptogyrin/SNG-1::GFP, concentrate at the presynaptic domain. SVPs are transported throughout the dendrite and distal axon but do not accumulate there^45^ (Fig. 1b).

From an unbiased forward genetic screen we isolated a mutant, *wy743,* with an abnormal distribution of synaptogyrin/SNG-1::GFP in DA9. Two ectopic accumulations of synaptogyrin/SNG-1::GFP puncta are the defining feature of the *wy743* phenotype: one at the tip of the axon, and a second accumulation at the tip of the dendrite (Fig. 1c–e). In addition, dendrite length is reduced in *wy743* mutants to about 63% that of wt (Fig. 1f). SVP accumulations were also observed with another SVP marker, GFP::RAB-3 (Supplementary Fig. 1). The *wy743* phenotype is also manifested in other parts of the nervous system, including the DB neurons (Supplementary Fig. 2).

To learn whether the *wy743* defects are restricted to SVPs, we examined the localization of mitochondria. In wt controls, the mitochondrial marker TOMM-20::YFP is evenly distributed into small puncta along the dendrite (Fig. 1g). These puncta are displaced to the tip of the dendrite in *wy743* mutants, leading to an ectopic accumulation (Fig. 1h). We conclude that *wy743* affects different types of neuronal cargoes in several classes of *C. elegans* neurons.

To test whether the axonal SVP accumulations represent cargo sorting defects, we generated *wy743; unc-104(e1265)* double mutants. In *unc-104* mutants SVPs are depleted from the axon^46^ (Supplementary Fig. 1E). No axonal SVPs were detected in *wy743; unc-104(e1265)* double mutants (Supplementary Fig. 1F,H), suggesting that axonal cargo is not mis-sorted in *wy743* mutants. Since SVPs are already abundant in the dendrite of *unc-104* single mutants^46^ (Supplementary Fig. 1E), it is not possible to conclude from this experiment whether dendritic SVP accumulations in *wy743* are due to sorting defects in dendrites. Instead, we conducted an analogous experiment with the mitochondrial marker TOMM-20::YFP. We found that the TRAK/Milton homologue, *trak-1*, is required for mitochondria to enter dendrites (Fig. 1i). In *wy743; trak-1(tm1572)* double mutants the TOMM-20::YFP signal was completely eliminated from the dendrite (Fig. 1j). These results suggest that neither the axonal nor the dendritic accumulations in *wy743* mutants occur as a result of cargo missorting.

### *wy743* is an allele of dynein heavy chain *dhc-1*

**Figure 2:**
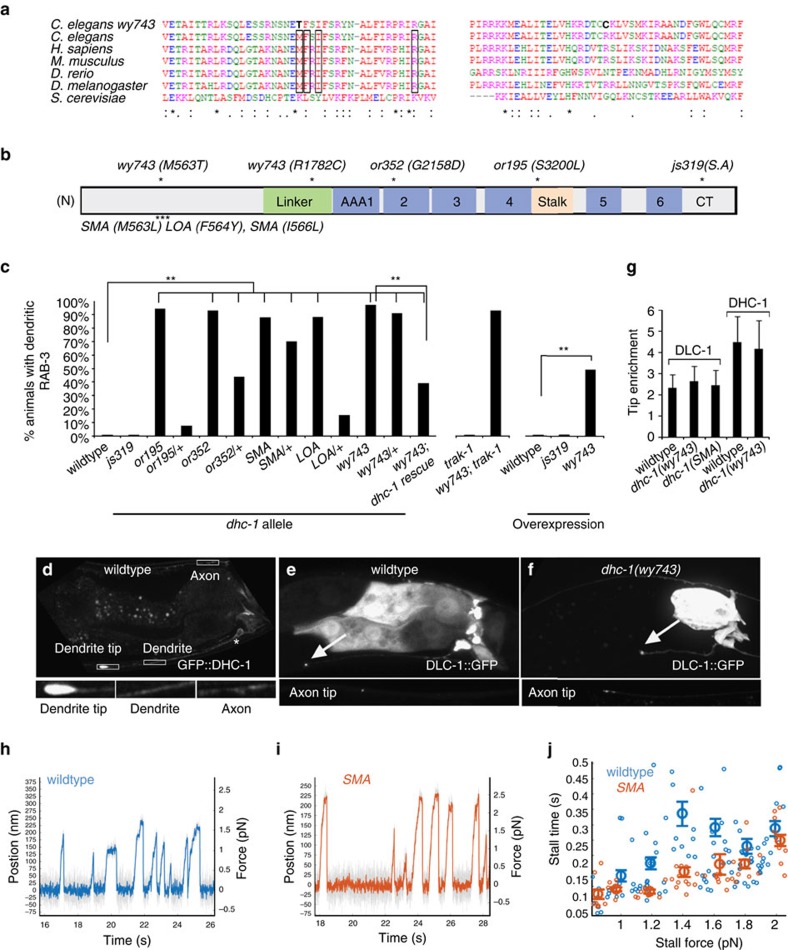
***wy743*** **is an allele of** ***dhc-1***. (**a**) Alignment of dynein heavy chain sequences encoding the N terminal (left) and linker domains (right), shows their high conservation. The mutated residues in *wy743* are highlighted. Boxes mark mutations that are associated with neuronal dysfunction in mice and humans. (**b**) Schematic drawing of DHC-1, with the conserved domains indicated. *C. elegans* mutations are indicated above, and the mouse and human mutations are marked beneath the scheme. (**c**) Quantification of dendritic SVP accumulations in *dhc-1* mutant alleles and transgenic rescue with genomic *dhc-1* fragment. L4 and young adults were examined on a wide-field microscope, and any worm with a GFP::RAB-3 signal at the tip of the denrite was scored as positive. *n*=27–60, **P<0.01, *χ*^2^-test. (**d**) Subcellular localization of a rescuing GFP::DHC-1 transgene expressed specifically in DA9. (**e**) DLC-1::GFP is also enriched at the dendrite tip and can be detected at the axon tip. (**f**) In *dhc-1(wy743)* mutants, the distribution of DLC-1::GFP is similar to wt. (**g**) Dynein tip enrichment was quantified as the ratio between GFP::DHC-1 or DLC-1::GFP in the tip and the entire dendrite, and normalized to a soluble mCherry marker. *n*=17–23. H,I. Representative stall-force traces for wildtype (**h**) and *SMA* (**i**) dynein immunoprecipitated with a FLAG tag from COS-7 cells. (**j**) Plot of stall-force vs. stall time showing that *SMA* dynein is less processive than wildtype *n*=270 (control) and 220 (*SMA*) from two independent experiments. *P*<0.01 for the range between 0.2 and 0.6 s, ANOVA. Note that the range of forces observed is consistent with 1–2 motors. SA, splice acceptor.

We mapped *wy743* to two substitutions in conserved residues of DHC-1. M563T affects the N-tail domain, and R1782C is located in the linker domain (Fig. 2a,b). A M582L mutation in human DYNC1H1 leads to dominant SMA^32^,^33^. To gain insight into the cellular mechanisms that may be involved in SMA we engineered the mutation into *C. elegans dhc-1* using CRISPR–Cas9 (ref. 47). The allele, designated *dhc-1(SMA),* resulted in a similar phenotype to *wy743* (Fig. 2c). To further test the link between *C. elegans dhc-1* mutations and neuronal dysfunction in other systems, we generated a worm *LOA* allele^48^. The mouse *LOA* mutation reduces dynein complex assembly and affinity to MTs during ATP hydrolysis^34^,^49^. *dhc-1(LOA)* worms showed cargo accumulations reminiscent of the *SMA* and *wy743* alleles (Fig. 2c).

To probe the mechanism leading to neuronal disease and to the phenotypes we observe in DA9, we engineered the *SMA* mutation into a rat DHC1-FLAG construct^50^, immunoprecipitated dynein from COS-7 cells onto beads and performed force measurements using an optical trap. Interestingly, although mutant and wt dynein produced similar stall forces, processivity under load was reduced in the mutant (Fig. 2h–j). Speed was not significantly affected (556±436 nm s^−1^ for wt, 491±314 nm s^−1^ for mutant, *P*=0.06). Consistently, the LOA allele also exhibits defects in processivity but not force generation^49^.

Four lines of evidence suggest that *wy743* impairs DHC-1 function. First, axonal SVP accumulation has been previously attributed to reduced dynein function in *C. elegans*^27^. Second, the *LOA* and *SMA* mutations phenocopy *wy743 in vivo* (Fig. 2c), and *in vitro* they reduce processivity (Fig. 2h–j)^49^. Third, two additional well-characterized *dhc-1* alleles, *or195* and *or352* (refs 51, 52, 53), which affect the stalk and ATPase domains, respectively, recapitulate the *wy743* SVP accumulation phenotype (Fig. 2c). A third allele *(js319)* that exhibits cargo accumulation at the axonal tip of touch receptor neurons^27^ showed axonal SVP accumulations in DA9 in 47% of animals. Interestingly, although *dhc-1(js319)* shows defects in SVP trafficking in DA9 (ref. 46), it showed no dendritic accumulations. This suggests that the other alleles affect a function of *dhc-1* that is not impaired in the *js319* allele. Fourth, we could partially rescue the *wy743* phenotype by introducing a transgene containing only the *dhc-1* genomic region (Fig. 2c).

To further probe the genetic nature of the alleles that show dendritic accumulations, we examined heterozygous animals. The resulting phenotypes range from fully penetrant defects (*wy743)* to no phenotype (*or195)*, suggesting that dendritic accumulations are not always a dominant phenotype. Overexpression of the *wy743* allele, but not wt or *js319*, resulted in dendritic accumulations that mimic the mutant phenotype (Fig. 2c). We conclude that *wy743* is a novel, dominant-negative allele of *dhc-1.*

We examined the subcellular localization of DHC-1 with a GFP::DHC-1 transgene expressed in DA9, and found that it is enriched at the dendrite terminal (Fig. 2d,g). We also monitored the localization of dynein light chain/DLC-1::GFP, a non-motor subunit, which depends on endogenous DHC-1's motor activity for its distribution, and is therefore a more reliable marker. As expected from a non-motor subunit, DLC-1::GFP showed more diffuse staining than GFP::DHC-1 but was still enriched at the dendrite tip in 85% of the animals (Fig. 2e,g). Axon tip enrichment was also frequently observed, consistent with reports from mammalian neurons^29^. These results indicate that a dynein pool exists at the dendrite tip, where defects arise in the mutant. DLC-1::GFP was not mislocalized in *dhc-1(wy743)* or *dhc-1(SMA)* mutants (Fig. 2f,g), suggesting that these alleles do not affect dynein's localization.

### Cargo accumulation is not caused by transport defects

Accumulated cargo in the distal axon in *dhc-1* mutants was attributed to transport defects, as axonal MTs are plus-end-out^27^. In contrast, as 90% of dendritic MTs in DA9 are minus-end-out^54^, accumulations in dendrites are inconsistent with impaired minus-end-directed motor activity.

**Figure 3:**
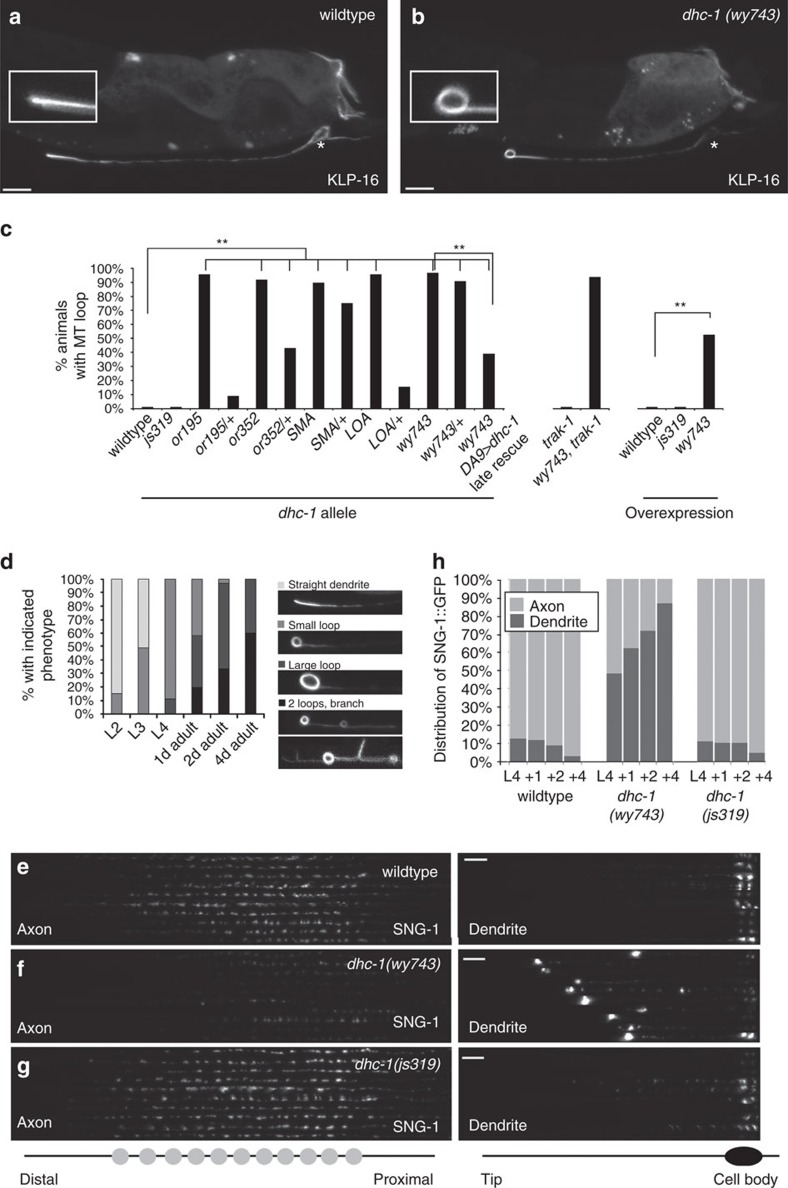
**Aberrant MT structures underlie SVP depletion from presynapses in** ***dhc-1*** **mutants**. (**a**,**b**) NCD/KLP-16::YFP fluorescence on dendritic MTs. KLP-16 is a minus-end-directed NCD-type motor, and is therefore enriched at the distal dendrite, reflecting the minus-end-out orientation of dendritic MTs. In a wt dendrite (**a**), MTs are straight. In *dhc-1(wy743)* (**b**), MTs show a prominent loop at the tip of the dendrite. (**c**) Quantification of dendritic MT loops in different *dhc-1* alleles and DA9 specific rescue. *n*=32–89 per genotype, **P<0.01. (**d**) Time course of MT morphological defects in *dhc-1(wy743)*. (**e**–**g**) Alignments of axons (left) and dendrites (right) taken from synaptogirin/SNG-1::GFP expressing worms at adult day 4 under identical settings. In *dhc-1(wy743),* the dendritic accumulations correlate with a depletion of SVPs from the presynapses. (**h**) Quantification of synaptogirin/SNG-1::GFP fluorescence in L4 through adult day 4 worms. *dhc-1(wy743)* gradually lose their synaptic SVPs as they accumulate in the dendrite. *dhc-1(js319)*, similar to wt, maintains the dendrite/axon SNG-1::GFP ratio.

We asked whether mitochondria mislocalization could underlie other defects in *dhc-1(wy743),* but found that *trak-1* mutants had no SVP accumulations, and *trak-1* did not suppress the *dhc-1(wy743)* SVP accumulations (Fig. 2c) or MT phenotypes (Fig. 3c).

One possibility is that *dhc-1* mutations cause MT polarity defects that lead to the dendritic phenotypes^55^. However, examination of MT polarity markers KLP-16::YFP and EBP-2::GFP (ref. 54) did not reveal any polarity defects (Figs 3 and 6).

Another possibility is that *wy743, LOA, SMA, or195* and *or352* are neomorphic alleles that bias DHC-1 dependent trafficking towards the MT minus-end. To investigate the relationship between transport defects and the accumulations, we imaged SVP trafficking in *dhc-1(wy743)* mutants and measured transport parameters on kymographs (Supplementary Fig. 3B). As a control we used wt animals and *dhc-1(js319)* mutants, in which trafficking is affected but no dendritic accumulations arise.

Retrograde speed in the axon, where MT are plus-end-out, was modestly decreased in *dhc-1(js319)* mutants compared to wt, and it was further decreased in *dhc-1(wy743)* (Supplementary Fig. 3C). Since the MTs in the dendrites are minus-end distal, anterograde SVP movements are likely mediated by dynein. Consistently, anterograde speed was reduced in the dendrites of *dhc-1(wy743)* and *dhc-1(js319)* compared to wt (Supplementary Fig. 3C). This indicates that changes in speed do not account for the accumulated dendritic cargo.

In the dendrite, we observed a similar small bias in directionality towards the minus end in both mutant alleles compared to wt (Supplementary Fig. 3E). Since *dhc-1(js319)* has no accumulations, we conclude that changes in this parameter are also not responsible for the *dhc-1(wy743)* phenotype. Likewise, run length decreases were apparent in both mutants (Supplementary Fig. 3D). Together, these results indicate that transport towards the tip is reduced in both alleles. This decrease is inconsistent with the cargo buildup that occurs only in *wy743*. Hence, *wy743,* and by inference from the steady-state phenotype, the *LOA, SMA*, *or195* and *or352* alleles, may also affect a transport-independent function of DHC-1.

### MT defects correlate with accumulated cargo in *dhc-1* mutants

We next examined axonal MTs, labelled with GFP-tagged Ensconsin MT Binding domain (EMTB::GFP) and GFP::TBA-1/α-Tubulin, and could not detect a difference between wt and *dhc-*1 mutants (Supplementary Fig. 4A–D). We then visualized dendritic MTs with the NCD-type minus-end-directed kinesin KLP-16::YFP, which is distributed on MTs in a distal to proximal gradient, causing it to be enriched at the dendrite tip^54^. In contrast to wt, *dhc-1(wy743)* mutants showed a prominent MT loop structure at the tip of the dendrite (Fig. 3a,b). The *dhc-1* alleles *SMA*, LOA, or195 and *or352* showed a similar phenotype, which could be rescued cell autonomously (Fig. 3c). Additional MT markers (EMTB::GFP, GFP::TBA-1, and EBP-2::GFP) confirmed the presence of the loop at the dendrite terminal (Supplementary Fig. 4A–D).

We wondered whether MT looping and dendritic cargo accumulations could be related, since they arise at the same location. Consistently, the *dhc-1(js319)* allele, which shows only axonal cargo accumulation, did not show dendritic MT abnormalities (Fig. 3c). Furthermore, examination of the MT loop in various *dhc-1* heterozygous alleles revealed precisely the same spectrum of phenotypic penetrance as the SVP accumulation (Fig. 3c). These results indicate that MT defects in the dendrite correlate with cargo accumulation.

### DHC-1 continuously maintains cystoskeletal integrity

Next, we studied the temporal progression of MT defects. A small loop at the tip of the dendrite was apparent in 15% of L2 larvae, 49% in L3 and all animals by L4. The appearance of dendritic SVP accumulations followed a similar developmental profile (Supplementary Fig. 4E). Along with dendrite growth, the size of the loop increased (2.2±0.3 μm in 1 day old adults, Fig. 3d). Adult animals occasionally had additional smaller secondary loops along the dendrite. However, as these loops were infrequent, we focused almost all our subsequent analysis on the terminal loop. With aging, the loop at the tip often collapsed and gave rise to other aberrant structures such as small branches or bifurcations (Fig. 3d). These observations suggest that *dhc-1* is continuously required throughout dendrite growth. Consistently, cell-specific rescue of *wy743* was achieved with a transgene driven by the *Pitr-1* promoter (Fig. 3c), which is only expressed after the mid L4 stage. Ongoing requirement for *dhc-1* was also observed with the temperature-sensitive allele *or195*, which showed increased defects upon shift from 13 °C to 25 °C at the L4 stage (Supplementary Fig. 4F). We conclude that *dhc-1* is continuously required to maintain MT morphology and cargo distribution.

### MT phenotypes correlate with reduced availability of SVPs

The progressive aggravation of the dendritic MT phenotypes in *dhc-1(wy743)* raises the possibility that cargo distribution defects are likewise exacerbated with age. We followed the accumulation of SVPs in the dendrite, in parallel with their loss from their normal location in the axonal synaptic patch. In wt animals, 90% of synaptogyrin/SNG-1::GFP fluorescence was localized to the axon through the first 4 days of adulthood (Fig. 3e,f,h). In *dhc-1(wy743)*, 48% of synaptogyrin/SNG-1 fluorescence was localized to the dendrite in L4 animals. The accumulation of synaptogyrin/SNG-1::GFP in the dendrite increased over time, until at day 4, 85% of the signal was in the dendrite (Fig. 3f,h). The accumulation of SVPs in the dendrite was accompanied by their disappearance from the synaptic patch (compare Fig. 3e,f). Hence, ectopic cargo accumulations in *dhc-1(wy743)* reduce the availability of SVPs at presynapses. *dhc-1(js319)*, which shows normal MTs but defective transport, had a phenotype similar to wt (Fig. 3g,h). Thus, maintenance of an intact dendritic cytoskeleton by DHC-1 is critical for ensuring normal levels of SVPs at presynapses.

### DHC-1 maintains the spatial distribution of MTs

**Figure 4:**
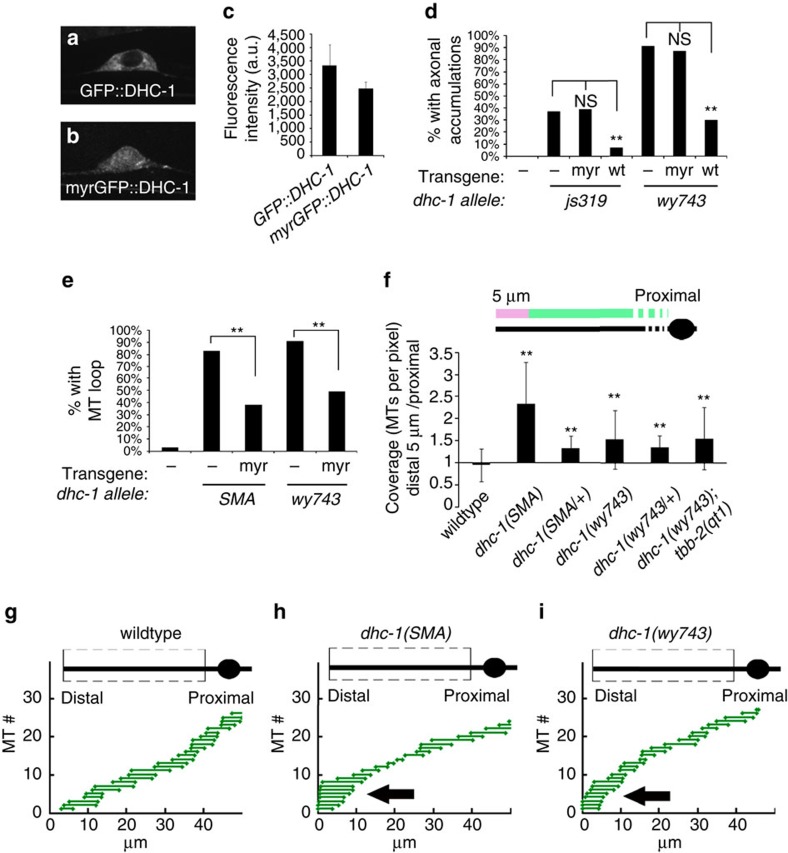
***dhc-1*** **cortical rescue and a distal shift in MT distribution in** ***dhc-1*** **mutants**. (**a**) GFP::DHC-1 is localized to the cytoplasm. (**b**) myrGFP::DHC-1 shows a distribution consistent with membrane localization. (**c**) Expression levels of GFP::DHC-1 and myrGFP::DHC-1 were quantified after imaging in identical settings. *n*=17 per genotype. (**d**) The myrGFP::DHC-1 construct does not rescue the accumulation of axonal SVPs (GFP::RAB-3). *n*=20–25, **P<0.05. (**e**) membrane tethered myrGFP::DHC-1 rescues MT loop formation in *dhc-1* mutants. *n*=28–45, **P<0.05. (**f**) Ratio between MT coverage (number of MTs per pixel) in the distal 5 μm of the dendrite to the rest of the dendrite. The results indicate that the distribution of MTs is shifted distally in *dhc-1* mutants. (**g**–**i**) Representations of MTs in the dendrites of indicated phenotypes. The representation is derived from a quantized GFP::TBA signal, where steps up indicate a MT start and steps down a MT end. MT ends were randomly assigned to the longest MT in the bundle for representation purposes. See Methods for details.

Besides the soluble dynein fraction, a cortically anchored dynein pool can transport MT polymers^41^ or enhance synaptic stability^38^,^41^. To ask whether immobilized DHC-1 could be mediating the effect on MTs in DA9, we injected a construct harbouring a myristoylated, GFP-tagged version of DHC-1 driven by a DA9 promoter into *dhc-1* mutants. Unlike GFP::DHC-1, which was cytoplasmic, the myritoylated construct showed a distribution that is consistent with membrane localization^56^ (Fig. 4a,b). Both constructs were expressed at comparable levels (Fig. 4c). We assayed the ability of both GFP::DHC-1 and myrGFP::DHC-1 to rescue axonal SVP accumulations, which are due to transport defect, in *js319* and *wy743* alleles, and observed no rescue with the myristoylated version (Fig. 4d). This suggests that while this construct may also generate a soluble DHC-1 pool that integrates into a dynein complex, it does not support efficient vesicular cargo transport. Despite this, the *myrGFP::dhc-1* construct rescued loop formation in *dhc-1(SMA)* and *dhc-1(wy743)* mutants (Fig. 4e). The rescue was incomplete, but comparable to the partial rescue obtained with non-myristoylated versions (Figs 2c and 3c), which is likely due to the dominant nature of the alleles and their putative effect on dynein complex stability^34^,^49^. This result suggests that the dynein pool which prevents MT loop formation is not moving on MTs.

To better understand the effect of DHC-1 on MT organization in the dendrite, we measured average MT length, coverage (MT numbers at any point along the dendrite) and spacing (distance between minus ends) using a recently established method^19^,^57^. MT length, coverage and spacing along the entire dendrite were not dramatically affected in *dhc-1* mutants (Supplementary Fig. 5). However, we noticed a significant distal shift in the distribution of MTs. We used the ratio between MT coverage at the distal dendrite (defined here as 5 μm, which is approximately the average MT length) and MT coverage at the rest of the dendrite to quantify this phenomenon. In wt, MT coverage at the distal dendrite was slightly lower than along the dendrite (Fig. 4f,g, Supplementary Fig. 5). In *dhc-1* mutants, MT coverage is mildly reduced along the dendrite, and a concomitant increase of MT coverage is seen at the distal end (Fig. 4f–i, Supplementary Fig. 5). A similar trend was observed for heterozygous *wy743* and *SMA* animals, in line with the dominant nature of these alleles. The skewed MT distribution suggests that *dhc-1* is required to exert a force directed towards the cell body on the minus-end-out MTs in the dendrite. When *dhc-1* function is compromised, the distribution of dendritic MTs is skewed distally, possibly by actin based forces^35^ or a MT motor^38^.

### DHC-1 acts on plus-end-out MTs to prevent loop formation

**Figure 5:**
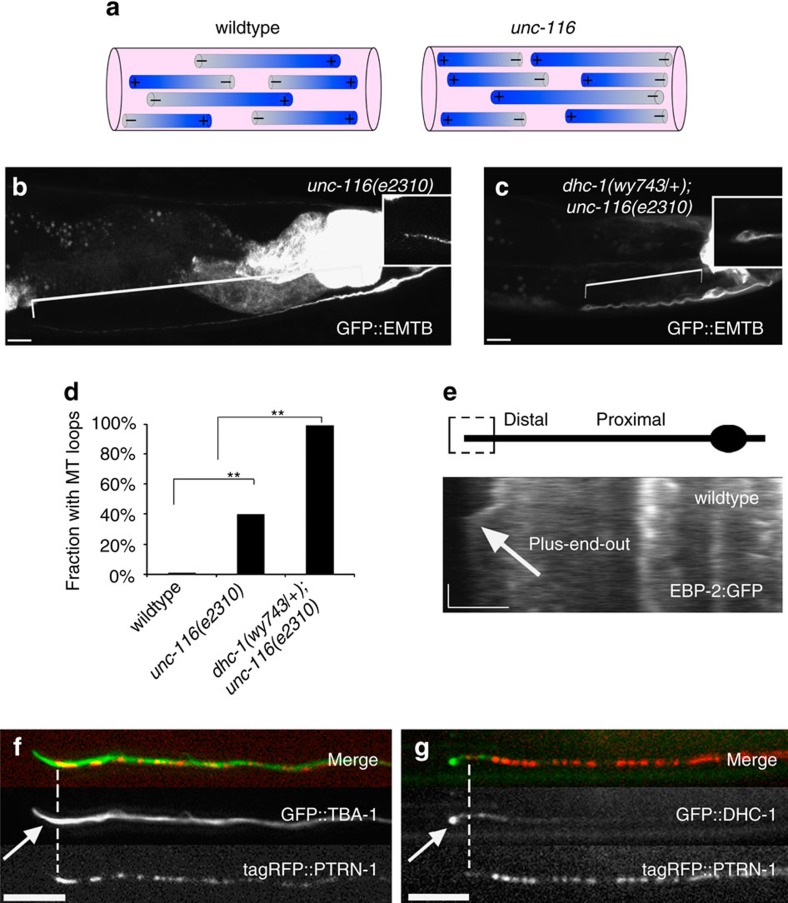
DHC-1 acts on plus-end-out MTs. (**a**) Scheme illustrating MT polarity in wt dendrites (mixed, but mostly minus-end-out) versus *unc-116(e2310)* dendrites (uniformly plus-end-out). (**b**) MTs, labelled by GFP::EMTB, in *unc-116(e2310)* mutant dendrites. (**c**) In *dhc-1(wy743/+); unc-116(e2310)* double mutants the terminal MT loop is apparent in all dendrites despite the plus-end-out orientation of MTs. (**d**) Quantification of MT loops in the indicated genotypes. *n*=25–31, **P<0.01. (**e**) A rare example of plus-end-out MT growth (labelled by EBP-2::GFP) at the tip of a wildtype dendrite. (**f**) tagRFP::PTRN-1 labels MT minus-ends. The presence of the MT marker GFP::TBA-1 after the distal-most tagRFP::PTRN-1 punctum indicates the presence of plus-end-out MTs at the dendrite tip. (**g**) GFP::DHC-1 accumulates distal to the last tagRFP::PTRN-1 punctum, suggesting that dynein is positioned in a location that allows it to interact with MT plus-ends.

The distal enrichment of MTs in *dhc-1* mutants may lead to looping. This model predicts that looping would depend on MT polarity, as in the dendrite dynein would push minus-end-out MTs towards the cell body and plus-end-out MTs outwards. We therefore examined *unc-116(e2310)*/kinesin-1 mutants, in which dendritic MT polarity is flipped to a plus-end-out configuration^54^. In this background, an anchored DHC-1 is expected to push MTs distally, and MTs should accumulate proximally in a *dhc-1* mutant. *dhc-1(wy743); unc-116(e2310)* double mutants were lethal, so we used *dhc-1(wy743/+); unc-116(e2310),* as *dhc-1(wy743/+)* heterozygotes still show the MT loop. The double mutant maintained the *unc-116*/kinesin-1 phenotype of reversed MT polarity (Supplementary Fig. 6A–C). Surprisingly, we found that in *unc-116* mutants, *dhc-1* was still required to prevent MT loop formation (Fig. 5a–d). This suggests that preventing MT looping by *dhc-1* does not require minus-end-out MTs. Since only minus-end-out MTs are expected to shift distally in *dhc-1* mutants, this result also suggests that the loops are not caused by the distal MT shift. Likewise, this reinforces the notion that transport defects do not account for the MT loops, as DHC-1 is expected to transport cargo towards the cell body in *unc-116(e2310)*. Interestingly, some MT loops that accumulated cargo were observed in *unc-116* single mutants (see Fig. 5d for MT loops). This effect is likely due to reduced levels of dynein at the dendrite tip in these mutants, as it does not move anterogradely on a plus-end-out array^54^, and would not be efficiently transported by mutant *unc-116*/kinesin-1 (refs 58, 59). Consistently, DLC-1::GFP was reduced, but not eliminated at the dendrite tip in *unc-116* mutants (Supplementary Fig. 6D–F).

Since these results suggest that plus-end-out MTs are mediating loop formation in *dhc-1* mutants, we asked whether such MTs are found at the dendrite tip. We examined MT polarity using GFP::EBP-2, which binds to the growing ends of MTs^60^. MTs at the dendrite tip were extremely static and rarely showed dynamic behaviour that would indicate their polarity. However, we could observe rare growth events, which were directed outwards (Fig. 5e). Plus-end-out growth at the dendrite tip was also observed in *efa-6* mutants (Supplementary Fig. 7C), which were reported to increase MT dynamics^61^.

We next examined MTs that were marked with GFP::TBA-1 and decorated with RFP::PTRN-1, which binds minus ends^62^,^63^,^64^. GFP::TBA-1 often extended beyond the distal-most RFP::PTRN-1 punctum, indicating the presence of plus-end-out MTs at the dendrite tip. Similarly, GFP::DHC-1, which accumulates at the tip of the dendrite, was often found beyond the distal-most RFP::PTRN-1 punctum (Fig. 5f,g).

Since plus-end-out MTs are sufficient for loop formation in *dhc-1(wy743/+); unc-116(e2310)* mutants, we wondered if the loops could also form at axonal tips, where MTs are oriented plus-end-out. No loops were detected in *dhc-1* mutants at the tip of the DA9 axon (Supplementary Fig. 4). However, in DA1, the anterior-most DA neuron, MT loops were present in *dhc-1(wy743)* mutants but not in wt (Supplementary Fig. 7A,B). This result confirms that plus-end-out MTs mediate the *dhc-1* phenotype.

### DHC-1 suppresses MT dynamics at the distal dendrite

**Figure 6:**
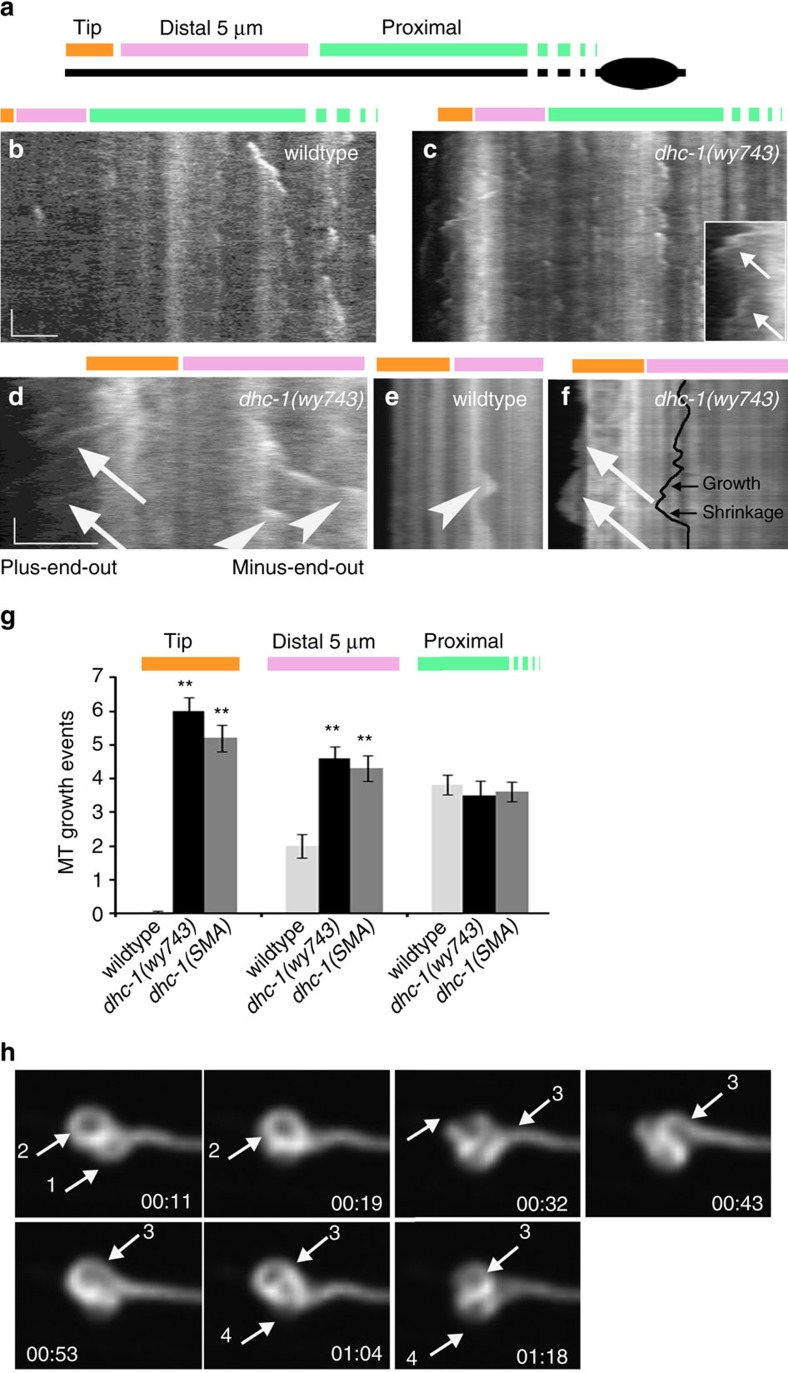
**Highly dynamic plus-end-out MTs at the tip of the dendrite in** ***dhc-1*****mutants**. (**a**) Scheme illustrating the division of the dendrite into three areas. (**b**) Kymograph from EBP-2::GFP movies of a wt L4 animal. MT growth is detected mostly near the cell body. (**c**) MT dynamics are increased in the distal dendrites of *dhc-1* mutants, particularly at the tip, where abundant plus-end-distal growth is observed (arrows). (**d**) An enlarged view of the distal dendrite from a *dhc-1(wy743)* animal. Abundant plus-end-out growth is detected (arrows). Before the tip, an elevation in the number of plus-end-proximal EBP-2::GFP comets (arrowheads) mirrors the increase in MT coverage of this area. (**e**) GFP::TBA-1 movie of wt reveals that growth of minus-end-out MTs (arrowhead) at the distal dendrite is coupled to shrinkage. No growth is observed at the tip. (**f**) Plus-end-distal growth of MTs (arrow) in the terminal loop of *dhc-1* mutants, visualized with GFP::TBA-1, is coupled to shrinkage. (**g**) The number of MT growth events (defined as either an EBP-2::GFP comet or a GFP::TBA-1 growth signal) in *dhc-1* mutants is mildly elevated in the distal dendrite and drastically elevated at the tip. *n*=24–37 movies per genotype. **P<0.01. See Supplementary Fig. 8 for quantifications of additional dynamic parameters. (**h**) Single frames from a GFP::TBA movie of *dhc-1(wy743)* showing the formation and collapse of four MT loops (arrows). The buckling of loop 3 and the collapse of loop 2 are particularly evident.

To understand the effect of DHC-1 on MT dynamics, we monitored the +TIP protein EBP-2::GFP (Supplementary Movies 1 and 2) and analysed the resulting kymographs^60^. As a second readout we used a GFP::TBA-1, which labels MTs uniformly. RFP::TBA-1 and EBP-2::GFP colocalize during MT growth^19^. Consistent with previous studies^19^,^54^, DA9 MTs showed a low frequency of EBP-2::GFP comets. We divided the analysis into three sections: the dendrite tip, the distal 5 μm and the proximal part extending to the cell body (Fig. 6a). In wt, MTs grew primarily at the proximal region. Fewer EBP-2::GFP comets were observed in the distal 5 μm, and almost none at the tip (Fig. 6b,g, Supplementary Movie 3). *dhc-1* mutants had normal MT dynamics near the cell body, mildly elevated dynamics at the distal 5 μm and a drastic increase at the tip (Fig. 6c,d,g, Supplementary Movies 4 and 5). Particularly striking was the increase in plus-end-out EBP-2::GFP comets at the tip (Fig. 6d), which contrasts with almost none in wt. Elevated MT coverage may explain the increase in EBP-2::GFP comets at the distal 5 μm but not at the tip, where the change is much higher than the ∼twofold increase in polymer numbers. Furthermore, plus-end-out MTs are predicted to be pushed towards the cell body in *dhc-1* mutants, suggesting that the increase in plus-end-out MT growth at the tip is not related to the skewed MT distribution.

Neuronal stress may increase MT dynamics through JNK signalling^65^. However, increased MT dynamics, looping and cargo accumulation were all maintained in *dhc-1(SMA); jnk-1(gk7)* double mutants (Supplementary Movie 8). These observations suggest that DHC-1 maintains MTs in a static state, and that this function is particularly relevant for plus-end-out MTs at the dendrite tip.

Observations with GFP::TBA-1 and EMTB::GFP revealed that excessive growth in *dhc-1* was accompanied by excessive shrinkage, resulting in repeated cycles of loop formation and collapse (Fig. 6f,h, Supplementary Movie 6).The speed and extent of MT growth and shrinkage were not altered in *dhc-1* mutants (Supplementary Fig. 8). In both wt and mutant, growth events were coupled to shrinkage, such that overall polymer content did not change significantly (Supplementary Fig. 8), consistent with the quantification of MT coverage (Supplementary Fig. 5). These results suggest that *dhc-1* is required to inhibit dynamic behaviour, not to prevent MT growth *per se*.

Loop formation in *dhc-1* mutants involved MT buckling and then twisting (Fig. 6h, Supplementary Fig. 9A, Supplementary Movies 6 and 7), which may result from excessive forces that push the MTs distally. Alternatively, plus-end-out MTs growing against a barrier could buckle and twist to form a loop when dynein is not found on the barrier to tether them^43^,^44^,^66^. To distinguish these possibilities we bleached a small region proximal to the loop, such that under the first model, the marked MTs should be observed moving into the loop as it collapses and reforms (Supplementary Fig. 9B). Despite abundant MT dynamics in the loop, we did not observe the marked MTs moving into the loop (Supplementary Fig. 9C). This indicates that loop formation and collapse do not involve MTs moving distally, consistent with a mechanism that acts locally.

### Dynamic MTs cause structural defects and cargo accumulation

**Figure 7:**
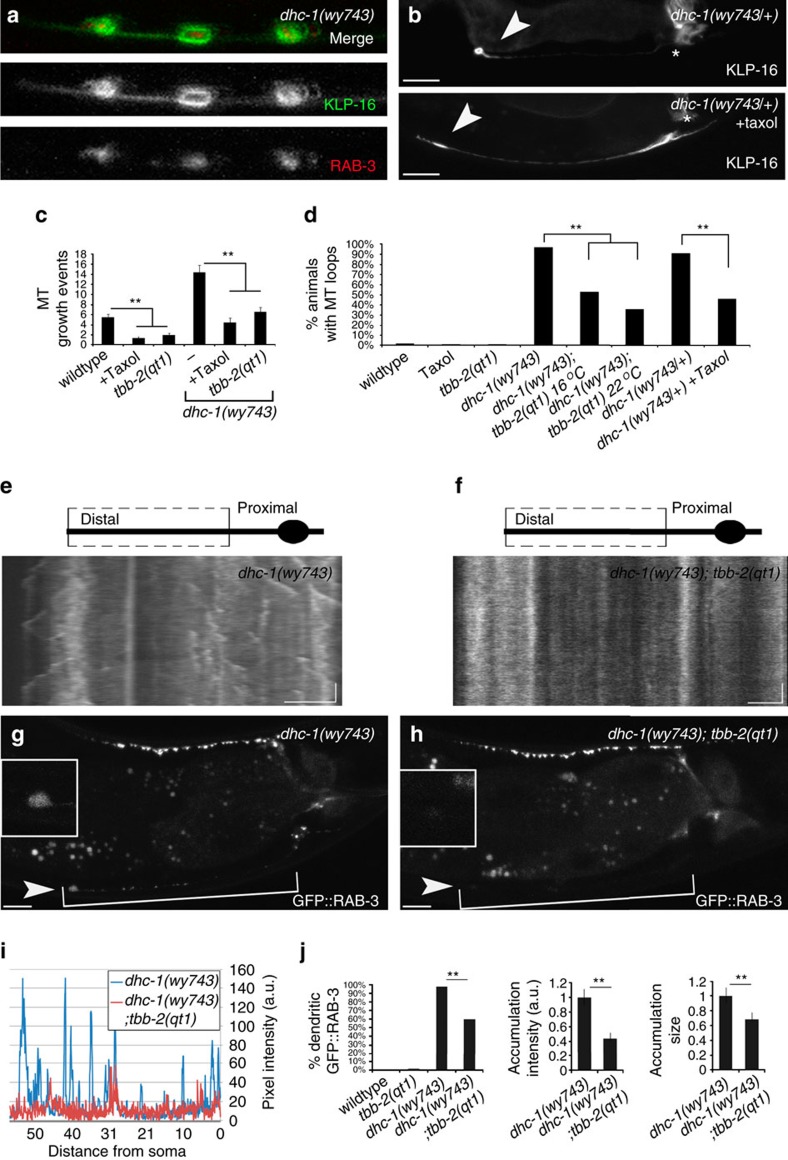
**MT instability is the cause of structural defects and cargo accumulation in** ***dhc-1*** **mutants**. (**a**) SVPs, marked by RAB-3::TdTomato (red) colocalize with secondary MT loops (NCD/KLP-16::YFP, green) in the dendrite of 3-day-old *dhc-1(wy743)* mutants. (**b**) MT stabilization by Taxol rescues loop formation in *dhc-1*(*wy743/+)* animals. (**c**) Quantification of MT dynamics in the presence of Taxol or the MT stabilizing mutation *tbb-2(qt1)* suggests that *tbb-2(qt1)* effectively stabilizes neuronal MTs in DA9. *n*=20–26 kymographs PER genotype, **P<0.01. (**d**) Quantification of the suppression of the terminal MT loop in *dhc-1* mutants by Taxol, or with the MT stabilizing mutation *tbb-2(qt1).*Note that some suppression occurs at the permissive temperature. This may reflect either a partial loss of function at the permissive temperature, or the fast-acting nature of this allele^52^. *n*=31–39 animals per genotype, **P<0.01. (**e**,**f**) Kymographs showing the stabilizing effect of *tbb-2(qt1)* on MTs in *dhc-1* mutants. *dhc-1(wy743)* mutants (**e**) show abundant MT growth, at the loop and the distal dendrite. This growth is suppressed in the double mutant *dhc-1(wy743); tbb-2(qt1)* (**f**). (**g**,**h**) Suppressing MT dynamics in *dhc-1* mutants abrogates cargo accumulation at the tip of the dendrite. *dhc-1(wy743)* mutants (**g**) show the typical SVP accumulation (markerd by GFP::RAB-3) at the tip of the dendrite. In *dhc-1(wy743); tbb-2(qt1)* double mutants (**h**) the accumulations are strongly reduced. (**i**) Fluorescence intensity profile of GFP::RAB-3 in the dendrite shows that SVP accumulations in *dhc-1(wy743)* are effectively eliminated by *tbb-2(qt1)* induced MT stabilization. (**j**) Quantifications of the frequency, size (normalized) and intensity of GFP::RAB-3 accumulations. The MT stabilizing mutation *tbb-2(qt1)* effectively reduces all these parameters in *dhc-1(wy743)* mutants. *n*=23–25 images per genotype, **P<0.01.

MT instability, loops and cargo accumulation in *dhc-1* mutants may arise independently, or be causally related. We noticed that in 3-day-old adult *dhc-1(wy743)* worms secondary loops accumulated SVPs, suggesting that MT abnormalities and cargo accumulation are related (Fig. 7a).

To test whether MT instability in *dhc-1*(wy743) leads to MT loops, we grew mutant animals at increasing concentrations of Taxol. Taxol efficiently suppressed EBP-2::GFP comets in wt or *dhc-1(wy743)* mutants (Fig. 7c). Concentrations up to 1 μM only rescued the loop in a small fraction of animals (15% rescue at 1 μM), whereas higher concentration were lethal to *dhc-1(wy743)* homozygotes (95% lethality at 2 μM). We therefore examined heterozygotes, which still show the mutant phenotype in almost all animals. At 2 μM taxol in the culture medium, 45% of the animals showed a straight dendrite, suggesting that excessive MT dynamics causes the loops (Fig. 7b,d).

We next examined a temperature-sensitive allele of β-tubulin, *tbb-2(qt1)*, in which an E198K substitution increases MT stability in *C. elegans* and *Aspergillus nidulans*^67^,^68^. The double mutant *dhc-1(wy743); tbb-2(qt1)* had significantly reduced MT dynamics compared to *dhc-1(wy743)* single mutants, indicating that *tbb-2(qt1)* can also stabilize neuronal MTs in *dhc-1* mutants (Fig. 7c,e,f).

The percentage of *dhc-1(wy743); tbb-2(qt1)* double mutants that showed the terminal loop was significantly reduced compared to *dhc-1(wy743)* single mutants (Fig. 7d), strengthening the hypothesis that unstable MTs cause the abnormal dendritic morphology. *tbb-2(qt1)* did not suppress the distal skew of MT density in *dhc-1* mutants (Fig. 4f), indicating that the role of DHC-1 in pushing MTs towards the cell body can be genetically uncoupled from its role in suppressing MT dynamics.

Lastly, we tested whether stabilizing MTs could also rescue the ectopic accumulation of SVPs in *dhc-1* mutants, and found that to be the case (Fig. 7g–j): the fraction of *dhc-1(wy743); tbb-2(qt1)* animals with accumulations was reduced by 40%, and in the animals that did show accumulations, the intensity of the GFP::RAB-3 at the dendrite was diminished compared to *dhc-1(wy743)* single mutants. Similarly, *dhc-1(wy743); tbb-2(qt1)* double mutants showed weaker accumulations of mitochondria at the tip of the dendrite compared to *dhc-1(wy743)* single mutants. This effect was accompanied by appearance of mitochondria at their normal location along the dendrite (Supplementary Fig. 10).

Together, these results suggest that DHC-1 prevents MTs at the dendrite tip from becoming excessively dynamic, and that MT structure and cargo distribution are aberrant when this stability is compromised.

## Discussion

Dynamic MT arrays are important during cell division, while stable MTs are required to support the long-range trafficking characteristic of neurons. Our results illustrate the consequences of a loss of MT stability in the dendrite. In *dhc-1* mutants, MTs become excessively dynamic, resulting in aberrant morphology and trapping of cargo at the dendrite tip at the expense of normal locations. Two trafficking-independent functions of DHC-1 shape the dendritic cytoskeleton: control of MT distribution and suppression of MT dynamics at the dendrite tip. We propose a model where the latter function is carried out by an interaction between anchored dynein and MT plus-ends.

Dynein's best described function is retrograde transport^25^,^26^,^27^,^28^,^29^. Although cargo mislocalizes in *dhc-1* alleles, several observations suggest that transport defects do not drive this phenotype: (1) dendritic MTs are minus-end-out; therefore, reducing minus-end-directed transport should lead to accumulations in the cell body, not the tip. (2) The *js319* allele is defective for SVP transport yet shows no tip accumulations. (3) Cargo accumulation can be rescued by MT stabilization. A related hypothesis, that dynein transports an unknown MT stabilizer to the dendrite tip is also inconsistent: (1) in this scenario, the *js319* allele would show MT loops. (2) Flipping MT polarity in *unc-116*/kinesin-1 mutants would eliminate the requirement for *dhc-1* in preventing MT loops. (3) The phenotype can be rescued by a myrisotylated DHC-1. In axons, forces exerted by anchored dynein push MTs towards their plus ends, and are opposed by myosin or kinesin dependent forces^35^,^36^,^37^,^38^. Our results suggest that a similar mechanism operates in the dendrite, accounting for the skewed MT density and the ∼twofold increase in MT dynamics at the distal 5 μm of *dhc-1* mutants. However, MT movement cannot explain the loop or cargo accumulation: it is inconsistent with the suppression of the loop by taxol. Furthermore, the fact that *tbb-2(qt1)* rescues loop formation and cargo accumulation but not the shifted MT distribution indicates that the latter defect does not account for former.

The rescue experiments with taxol and *tbb-2(qt1)* indicate that MT stabilization at the dendrite tip is required to prevent loop formation and cargo accumulation. Mechanistically, we favour a model where stabilization is carried out by immobile DHC-1 tethering the MT plus-ends at the dendrite tip. This is consistent with requirement for *dhc-1* on a plus-end-out MT array (Fig. 5, Supplementary Fig. 7), with the presence of non-dynamic plus-end-out MTs at the dendrite tip in wt (Fig. 5) and with the local increase in plus-end-out MT dynamics in *dhc-1* mutants (Fig. 4). Furthermore, this model is consistent with the accumulation of cargo at the dendrite tip despite reduced transport towards this location (Fig. 2, Supplementary Fig. 3), and with the rescue by myrGFP::DHC-1 (Fig. 4). Previous observations of tethering suggest that dynein interacts with MT plus-ends transiently to stabilize their dynamics^41^,^43^,^44^. Reduced processivity may affect tethering by reducing the time that dynein remains engaged with the plus end of the MT. Although dynein's localization suggests that this function is primarily carried by a pool at the tip of the dendrite, our data cannot exclude similar DHC-1 activity throughout the dendrite. In fact, this interpretation is consistent with the observed shift in MT density and with the low penetrance phenotype of secondary loops. Nevertheless, the appearance of all the related phenotypes at the dendrite tip suggests that the requirement for *dhc-1* dependent MT tethering and stabilization is most significant at this location.

The link between MT abnormalities, axonal transport and neuronal disease is becoming increasingly clear^30^,^31^. The *dhc-1(SMA)* allele suggests a possible cellular mechanism for disease-linked DYNC1H1 mutations: in the absence of MT tethering/stabilization, excessively dynamic MTs may impair the distribution of neuronal cargo.

## Methods

### Imaging

Worms were paralyzed in 3 mM levamisole and mounted on 2% agar pads. For time lapse movies, worms were paralyzed in 0.4 mM levamisole for 15 min, then mounted in M9 buffer on 10% agarose pads.

Imaging was done on a Zeiss LSM710 scanning confocal microscope using a Plan-Apochromat 40 × 1.3 objective. For time lapse movies, a Zeiss Axio Observer equipped with a Plan-Apochromat 63 × 1.4 objective, a Yokogawa spinning disc and 488 nm laser line was used. The Mosaic system (Andor) was used for photobleaching with a 405 nm laser line. For SVP trafficking movies, we used streaming mode with 100 ms exposure. For MT dynamics, we used a frame rate of 7 s^−1^. Movies were 40 s to 1 min 30 s long depending on the experiment.

### Image analysis

For dynamic imaging, analysis was carried out using ImageJ. Images were first corrected for animal movement using the ImageJ plugin StackReg. The axon/dendrite was then traced and Kymographs were generated using the ‘reslice' function. For analysing MT dynamics at the tip of the dendrite, where the looping occurs, we only used animals where we could confidently follow the MTs (that is, determine proximal versus distal) throughout the loop.

Single events were traced manually on the kymographs and speed, run-length and directionality were measured using the ‘measure' function. We measured all visible events that were longer than 200 ms. Data was normalized by time and size of the imaged neurite segment. Quantifications of puncta size and intensity were carried out on images acquired under identical settings using the Analyze/Measure function in imageJ. Statistical tests and graphs were generated with Excel. Images were assembled into multi-panel figures using Photoshop.

Analysis of MT organization: this method is described in a separate manuscript^19^. Only a brief description is provided here. More detailed information is available upon request.

Worms that express GFP::TBA-1 and tagRFP::PTRN-1 specifically in DA9 were generated. The integration of GFP::TBA-1 into the MTs was measured by FRAP. tagRFP::PTRN-1 labels discrete puncta that correspond to MT minus-ends. A custom Matlab code then analyzes the images.

Briefly, the analysis consists of the following steps: (1) background subtraction. (2) A user dawn line is used to generate a mask of the neuronal process. The algorithm adjusts the mask using a reiterative process and sums all pixels intensities at each location along the mask. (3) Single MT fluorescence intensity extraction. In this step the code scans a range of possible intensities, and each one is used to quantize the GFP line scan. The intensity that minimizes the difference between the number of steps up on the quantized distribution and the number of observed tagRFP::PTRN-1 puncta is selected as the intensity of a single MT and is used to calculate the MT organization parameters. (4) Calculation of MT organization parameters: spacing of minus ends is calculated as the average distance between tagRFP::PTRN-1puncta in the line scan. Coverage is calculated by summing the GFP intensity along the process and dividing it by the previously calculated intensity of a single MT. The result is divided by the length of the scan to obtain the average MT numbers per pixel. Average MT length is obtained by dividing the total coverage by the number of MTs in the line scan.

All images analysed were manually verified and we discarded images with worm movement, a too dim signal or an inconsistent signal due to variations in distance of the process from the objective.

The method was validated by several means: (1) the results for the single MT intensity were similar to measurement of this parameter on kymographs, where the growth of a single MT allows to determine its fluorescence intensity. (2) Results for average MT length and coverage were in agreement with previously published EM reconstructions. (3) We performed an EM reconstruction of 10 μm in DA9 in one animal, as well as correlative fluorescence/EM analysis of 7.5 μm in another animal. In both cases the results of the fluorescent method were in good agreement with the EM reconstruction.

### Genetic screen and generation of *dhc-1(SMA) dhc-1(LOA)* alleles

All worm strains are listed in Supplementary Table 1. The *wy743* allele was isolated from a semiclonal F2 EMS screen of 2000 haploid genomes. SNIP-SNP mapping and rescue was performed using standard protocols. Whole genome sequencing was performed on Illumina Hi-seq platform and sequence analysis was carried out using CLC Genomics Workbench 5.

The *dhc-1(SMA)* and *dhc-1(LOA)* allele were generated by a CRISPR–Cas9-based methodology. We used two sgRNAs closely flanking the mutation, and a 200 bp oligo(LOA) or a PCR product (SMA) as a repair template. The presence of the mutation was verified by sequencing and the mutants were outcrossed four times to eliminate off-target mutations.

### Dynein force measurements

COS-7 cells were transfected with flag-tagged dynein heavy chain constructs using Fugene (Promega). Cell extracts were prepared by lysing cells on ice in BRB80 (80 mM PIPES, 1 mM MgCl2, 1 mM EGTA, pH 6.8) supplemented with 1% Triton-X, protease inhibitors (AEBSF, Aprotinin, Bestatin, E64 and Leupeptin), and 1 mM MgATP. Cell extract was centrifuged at 2,000 *g* for 5 min and 20,000*g* for 30 min. The supernatant was frozen in LN2 for further use. Anti-Flag coated beads (1.0 μm, carboxylated, Invitrogen) were incubated in cell extract for 60 min on ice, and then pelleted and resuspended in BRB80 two times before use.

Beads were diluted in motility buffer (BRB80 supplemented with 0.2 mg ml^−1^ BSA, 10 mM DTT, 10 mg ml^−1^ glucose, 1 μg ml^−1^ glucose oxidase, 0.5 μg ml^−1^ catalase, 2 mM ATP and 20 μM taxol) and added to a flow chamber. An optical trap was used to position beads over coverslip-attached MTs, and measure their forces and displacements using back-focal plane interferometry.

### DNA constructs and microinjection

Expression clones were made in the pSM vector, a derivative of pPD49.26 (A. Fire) with extra cloning sites (S. McCarroll and C.I. Bargmann, personal communication). Plasmids and sequences are available upon request. All constructs were sequenced prior to microinjection. TagRFP optimized for expression in worms was a gift from C. Frøkjaer-Jensen. The Rat DHC1::FLAG was a gift from Dr R. Vallee. Microinjection was carried out using standard procedures. For each injected construct, at least 2–3 lines were generated to control for variation in expression levels. *myrGFP::dhc-1* was constructed by recombineering a kanamycin_Pmig-13::myrGFP into WRM0639aB10, a *dhc-1* containing fosmid. Pmig-13::LoxP::STOP::LoxP::TagRFP::PTRN-1a(cDNA) was generated using Gibson cloning. Pitr-1::DLC-1::GFP and Pitr-1::3XGFP::EMTB were generated by standard restriction cloning. The SMA mutation was cloned into Rat DHC1::FLAG using mutated primers to amplify a ∼3.5 kb fragment centred around the mutation, which was then cloned back into the vector with FseI.

### Data availability

All strains and plasmids and data generated in this study are available from the authors.

## Additional information

**How to cite this article:** Yogev, S. *et al*. Local inhibition of microtubule dynamics by dynein is required for neuronal cargo distribution. *Nat. Commun.* **8**, 15063 doi: 10.1038/ncomms15063 (2017).

**Publisher's note:** Springer Nature remains neutral with regard to jurisdictional claims in published maps and institutional affiliations.

## Supplementary Material

Supplementary Information Supplementary Figures and Supplementary Table

Supplementary Movie 1 EBP-2::GFP in a wildtype dendrite 220 frames acquired at150ms/frames and played at 20frames/sec.

Supplementary Movie 2 EBP-2::GFP in dhc-1(wy743) 220 frames acquired at150ms/frames and played at 20frames/sec.

Supplementary Movie 3 zoom-in on a wildtype dendrite tip 220 frames acquired at150ms/frames and played at 20frames/sec.

Supplementary Movie 4 zoom-in on a dhc-1(wy743) dendrite tip 220 frames acquired at150ms/frames and played at 20frames/sec.

Supplementary Movie 5 zoom-in on a dhc-1(wy743) dendrite tip with photobleaching after 1st frame 450 frames acquired at190ms/frames and played at 30frames/sec. Photobleaching after the 1st frame is used to reduce some of the diffuse background.

Supplementary Movie 6 wildtype and dhc-1(wy743) dendrite tips labeled with GFP::TBA-1 316 frames acquired at 190ms/frame and played at 30frames/sec. dhc-1 mutants show repeated formation and collapse of MT loops.

Supplementary Movie 7 GFP::EMTB labeled MT loop Short section (24 frames) showing loops formation by buckling and twisting. Acquired at 150ms/frame and played at 20frames/sec.

Supplementary Movie 8 GFP::TBA-1 in dhc-1(SMA); jnk-1(gk7) mutants 316 frames acquired at 190ms/frame and played at 30frames/sec. jnk-1(gk-7) does not modify the dhc-1 phenotype.
