# Supplementary material for: Local inhibition of microtubule dynamics by dynein is required for neuronal cargo distribution
Source: Nat Commun. 2017 Apr 13;8:15063. doi: 10.1038/ncomms15063 (PMC5399302; doi:10.1038/ncomms15063)
Supplement: Supplementary Information — Supplementary Figures and Supplementary Table [file ncomms15063-s1.pdf]

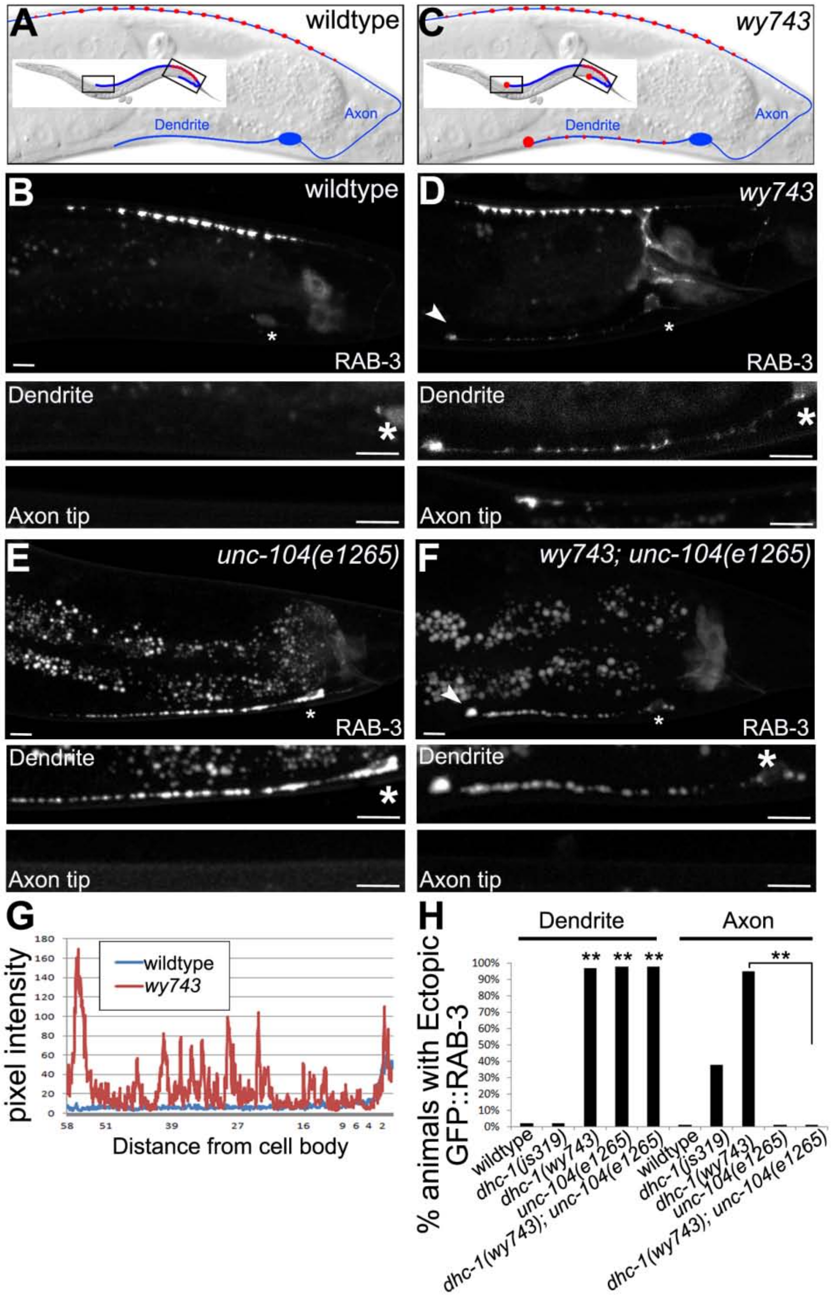

Supplementary Figure 1: GFP::RAB-3 accumulates at the tips of DA9 processes in *wy743* mutants

A. Schematic diagrams of the DA9 neuron in a wt animal. Presynapses are marked in red. B. Confocal micrograph of a wt young adult showing SVPs labeled by GFP::RAB-3. The cell body is marked by an asterisk. Lower panels show magnifications of the dendrite and the axon tip, which is out of the field of view in the upper panel. C. Schematics of the ectopic SVP accumulations at the DA9 dendrite of *wy743* mutants. D. Confocal micrographs of GFP::RAB-3 expressed in DA9 in *wy743* mutants. The presynaptic sites are correctly positioned, but additional ectopic fluorescence is observed in the dendrite, particularly at the distal tip. Lower panels show magnified views of SVP accumulation at the dendrite and axon tip. E. In *unc-104(e1265)* mutants SVPs are depleted from the axon and accumulate in the cell body and dendrite. F. *unc-104* suppresses the axonal SVP accumulations in *wy743*, suggesting cargo sorting to the axon occurs correctly. The dendritic phenotype is not suppressed, as SVPs are already present in *unc-104* single mutants. G. Intensity plot of GFP::RAB-3 fluorescence in wt (blue) and *wy743* (red) dendrites from panels B and C, showing the enrichment of SVPs at the distal tip. H. Quantification of dendritic and axonal GFP::RAB-3 accumulations. n=33-46 animals/genotype, **p<0.01, Chi-square test.

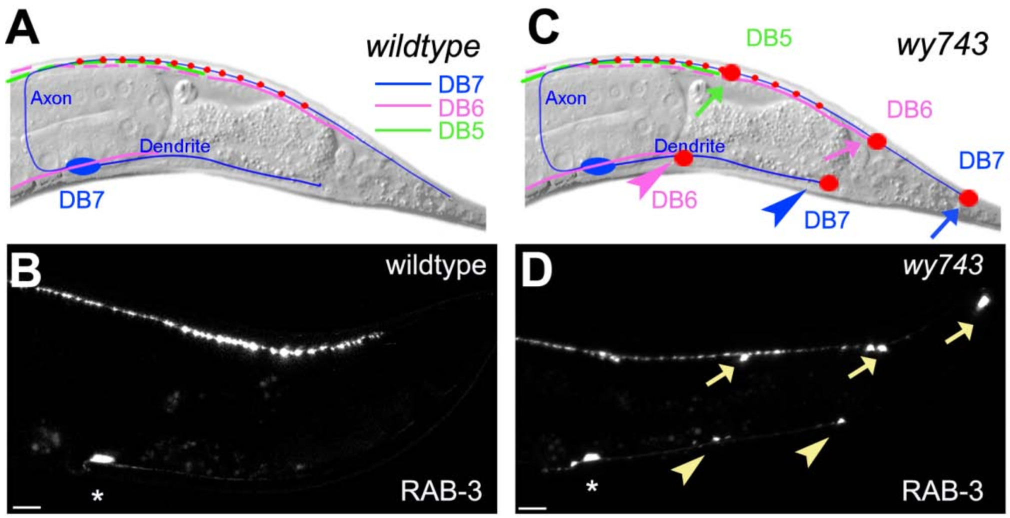

Supplementary Figure 2: SVP accumulations in DB neurons of *wy743* mutants

A. Schematic of the DB7 motor neuron (blue) expressing GFP::RAB-3 from the *unc-129* promoter, which is expressed in all DB neurons. Presynapses are marked in red. The processes of DB5 and DB6, which overlap with those of DB7, are shown in green (DB5) and pink (DB6). B. Confocal micrograph of a wt animal showing the localization of GFP::RAB-3 in DB7. No SVP are detected in the dendrite and the distal axon. The synaptic regions of DB5 and DB6 are not in the field of view. C and D. Schematic and confocal micrograph showing the distribution of GFP::RAB-3 in *wy743* mutants. In addition to its enrichment in presynapses, strong GFP::RAB-3 is also present at the tips of the dendrites and axons. Note that whereas only DB7 can be seen in the wt because of the restriction of GFP::RAB-3 to synaptic regions, the mutant has in the field of view additional fluorescence from the dendrite and axon tips of DB5 and DB6 neurons. Arrowheads and arrows mark the ectopic accumulations at the dendrite and axon tips, respectively. Scale bar is 5 microns.

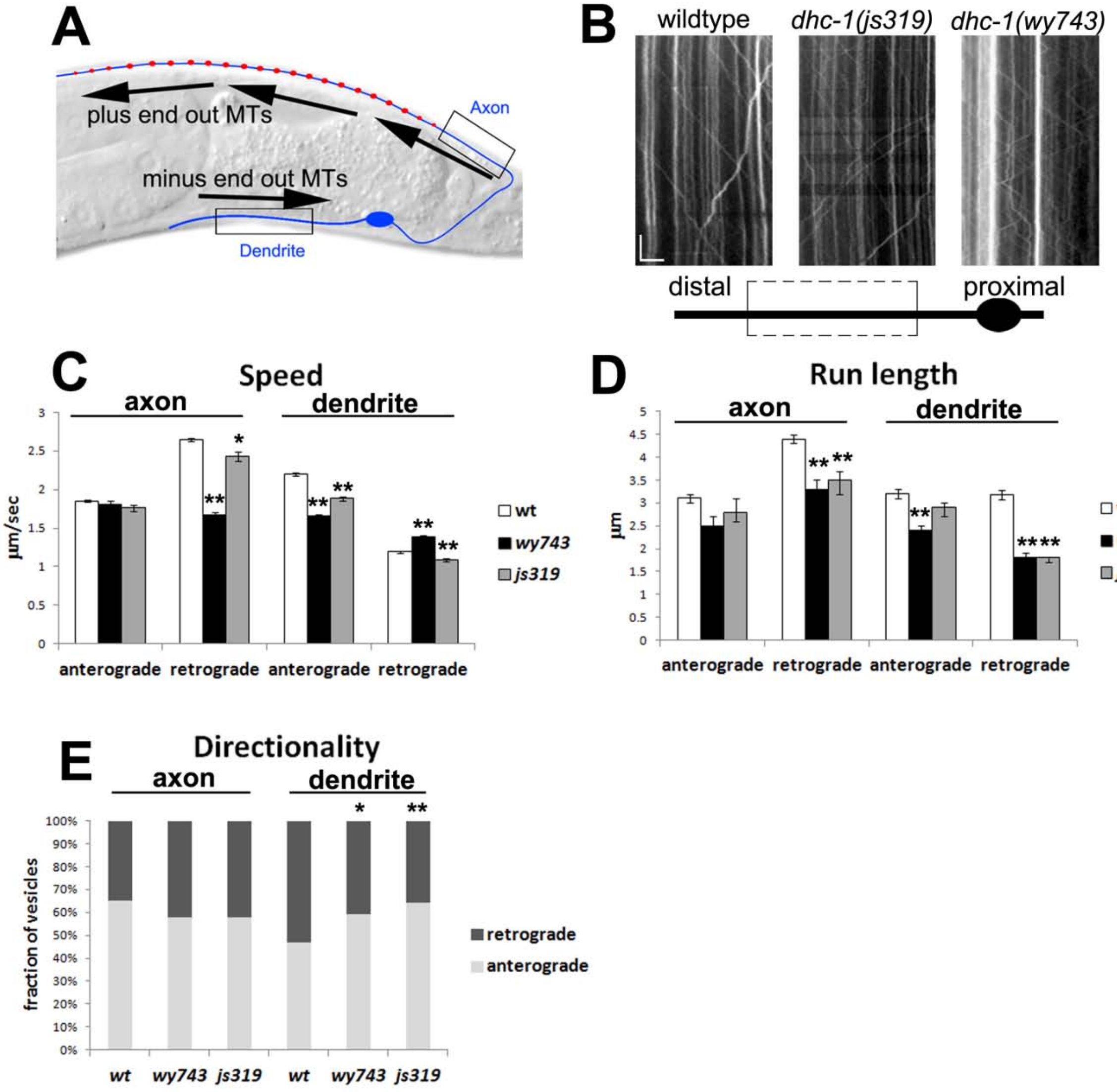

Supplementary Figure 3: Trafficking defects do not account for the dendritic vesicle accumulations in *dhc-1(wy743)* mutants

A. Schematics of MT polarity in DA9. Red circles denote the presynapses, and arrows show the polarity of MTs. MT polarity in the axon is plus end out, suggesting that the accumulation of cargo there in *dhc-1* mutants stems from a defect in retrograde trafficking. MT polarity in the dendrite is largely minus end out, making the accumulation of SVPs there in *dhc*-1 mutants inconsistent with a defect in retrograde transport. Boxes indicate where imaging of cargo transport was performed. B. Example kymographs from dynamic imaging movies of wt, *dhc-1(js319)* and *dhc-1(wy743)* dendrites, performed as previously published[48] and described in the methods section. The scheme on the bottom indicates the location of the imaged area with respect to the cell body and dendrite tip. Scale bar is 5 seconds and 5 microns. The *js319* allele was chosen for this analysis because it does not show the vesicle accumulations at the tips of the dendrite. C-E: dynamic parameters of synaptogyrin/SNG-1::GFP transport. *=p<0.05 and **=p<0.01. C. Speed measurement of SVPs reveal that the wy743 allele compromises DHC-1 function , as both retrograde speed in the axon and anterograde speed in the dendrite are reduced. D. Both *dhc-1(js319)* and *dhc-1(wy743)* show reduced cargo run-length. E. The directionality (anterograde vs retrograde) of moving SVPs suggests that more vesicles are moving towards the dendrite tip in *dhc-1(js319)* compared to *dhc-1(wy743)*. Note that all parameters suggest that in the allele that does not show accumulations *(js319),* there is more transport towards the dendrite tip than in the allele that does show accumulations *(wy743)*. This indicates that a bias in the transport towards the dendrite tip is not responsible for the accumulations.

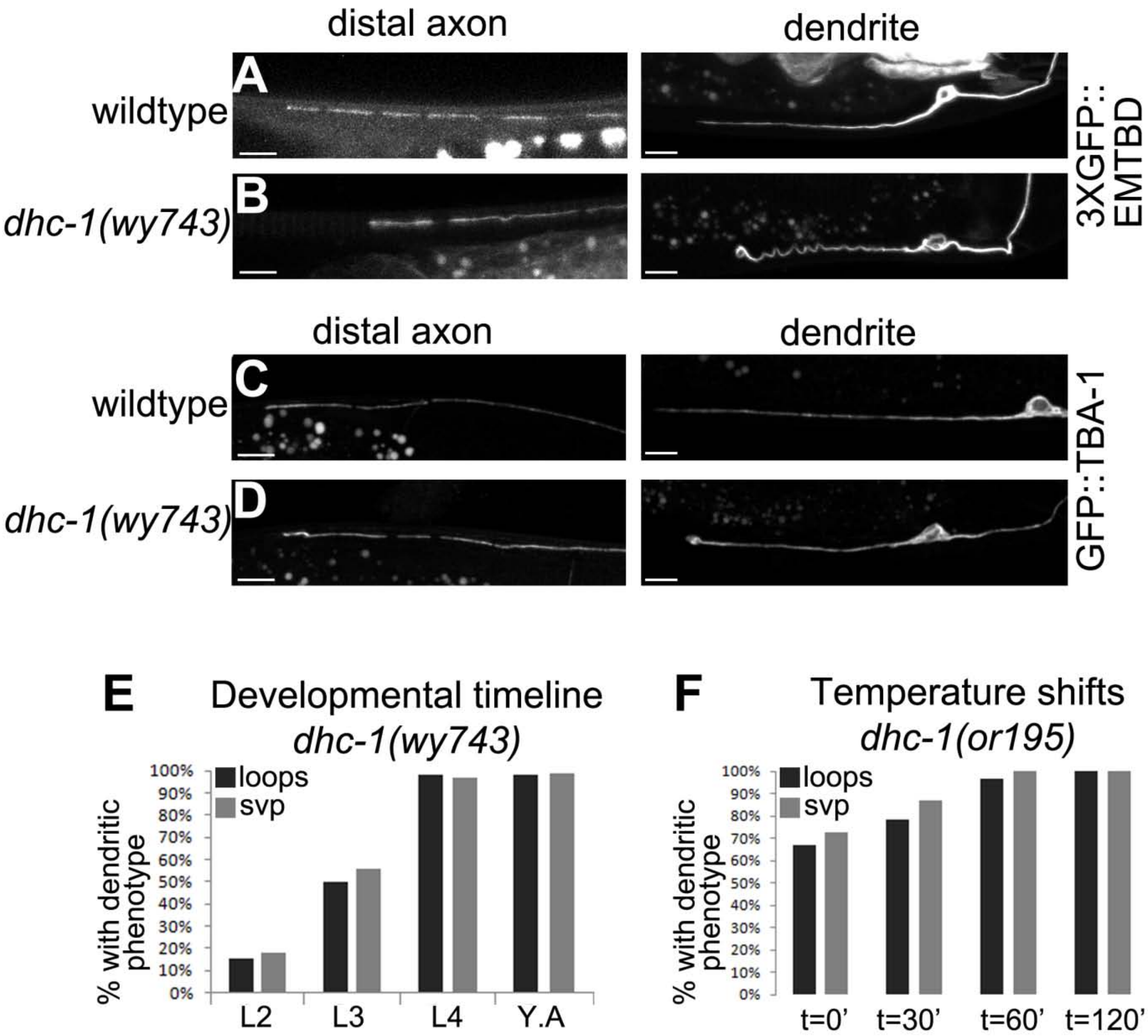

Supplementary Figure 4: MT defects in DA9 in *dhc-1(wy743)* mutants are restricted to the dendrite and increase during development

A. The tip of the axon (left panel) and the dendrite (right panel) in a wt animal expressing 3XGFP::EMTB specifically in DA9 with the *itr-1* promoter. Scale bar is 5 microns. Both the tip of the axon and tip of the dendrite are straight. B. in *dhc-1(wy743)* mutants, MTs at the tip of the axon appear similar to wt. In the dendrite, the aberrant MT loop which can be observed with KLP-16::YFP is also detected with 3XGFP::EMTB. C. GFP::TBA-1 labels axonal MTs. The dendrite tip is straight (right panel) as seen with other markers. D. In *dhc-1(wy743),* no abnormalities are seen at the position of the DA9 axon tip (left panel). The left panel shows TBA-1::GFP decorating the MT loop at the tip of the dendrite, as seen with KLP-16::YFP and 3XGFP::EMTB. E. SVP accumulations and MT loops in *dhc-1(wy743)* mutants appear concomitantly during development. n=20-42 animals per stage. F. *dhc-1(or195)* animals were grown at 13°C and moved to 25°C as young adults. The progression of the phenotypes was observed over 2 hours. n=33-51 animals per time point.

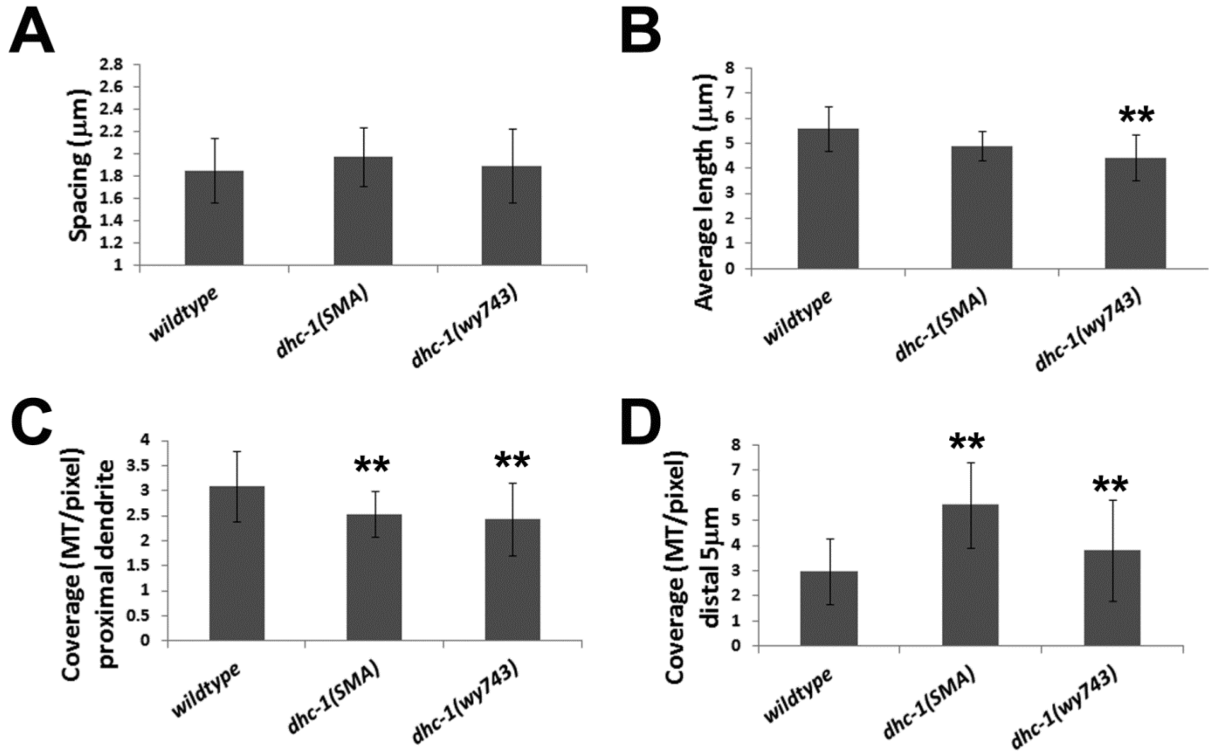

Supplementary Figure 5: Parameters of MT organization in the dendrites of wildtype and *dhc-1* mutants reveal a distal shift in MT density

MT organization was analyzed in the DA9 dendrite. Error bars represent standard deviation, **p<0.01, n=19-41, t-test. A. Spacing between adjacent minus ends. B. Average MT length is slightly reduced in *dhc-1(wy743)* but not in *dhc-1(SMA)* mutants. C. MT coverage along the dendrite is reduced in *dhc-1* mutants, consistent with a migration of some of the MTs towards the dendrite tip. D. Higher numbers of MTs per pixel in the distal dendrite of *dhc-1* mutants correlate with the reduced coverage along the dendrite. This indicates the MT mass has shifted distally in these mutants.

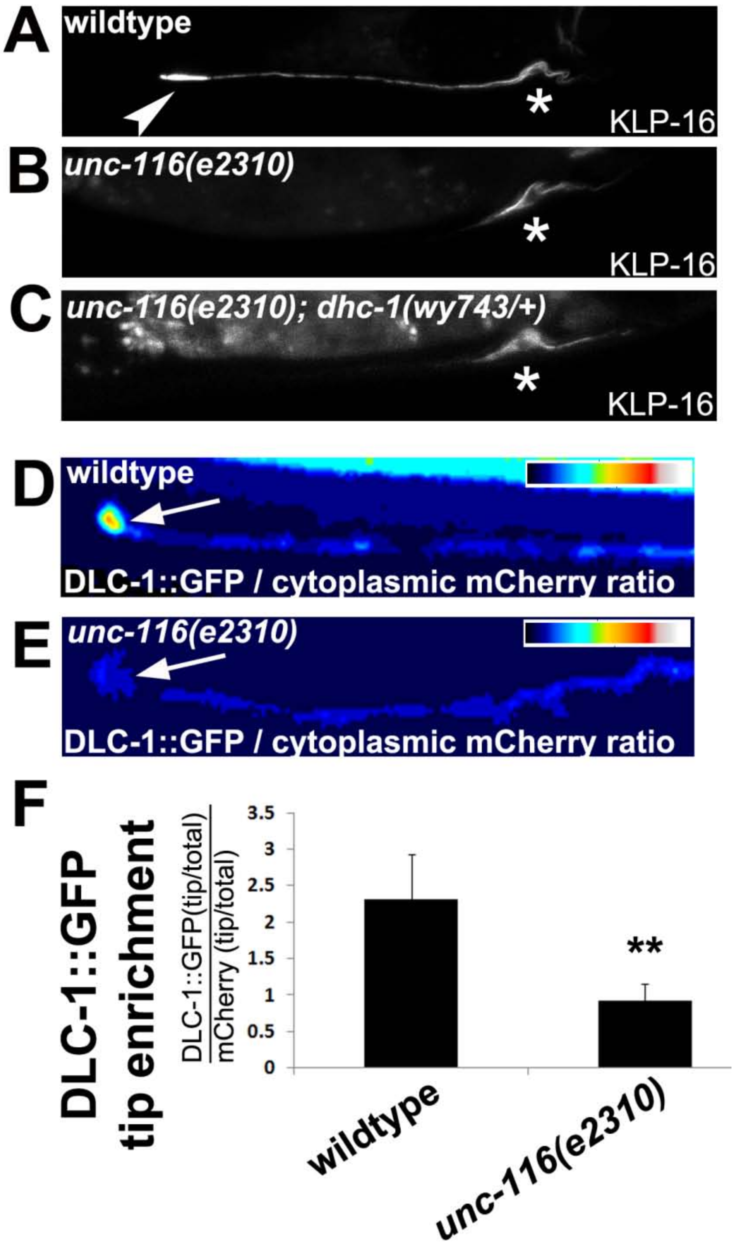

Supplementary Figure 6: reversed MT polarity in *dhc-1(wy743/+); unc-116(e2310)* mutants and reduced DLC-1::GFP at the dendrite tip in *unc-116* mutants

A. In a wt dendrite, the minus-end-directed NCD/KLP-16::YFP motor accumulates at the tip, confirming that dendritic MTs in DA9 are oriented plus-end-proximal. B. *unc-116(e2310)* mutants reverse MT polarity in the dendrite, to assume a mostly plus-end-out array. This leads to the loss of KLP-16::YFP from the dendrite tip. C. *dhc-1(wy743/+)* does not suppress the reversed polarity phenotype of *unc-116(e2310)*, as KLP-16::YFP is still absent from the dendrite tip. D-F. DLC-1::GFP was imaged with a cytoplasmic marker (mCherry) for quantification of the local tip enrichment. D. Pseudo-colored image showing the fluorescence ratio of of DLC-1::GFP to mCherry. An enrichment of DLC-1::GFP is observed at the dendrite tip. E. In *unc-116(e2310)* the ratio of DLC-1::GFP to mCherry is uniform along the length of the dendrite, suggesting that DLC-1::GFP is reduced, but not eliminated from the dendrite tip. F. Quantification of the DLC-1::GFP tip enrichment, calculated as the fluorescence ratio between the tip and the rest of the dendrite, normalized by the mCherry tip enrichment. Error bars represent standard deviation, n=15-19, **p<0.01, t-test.

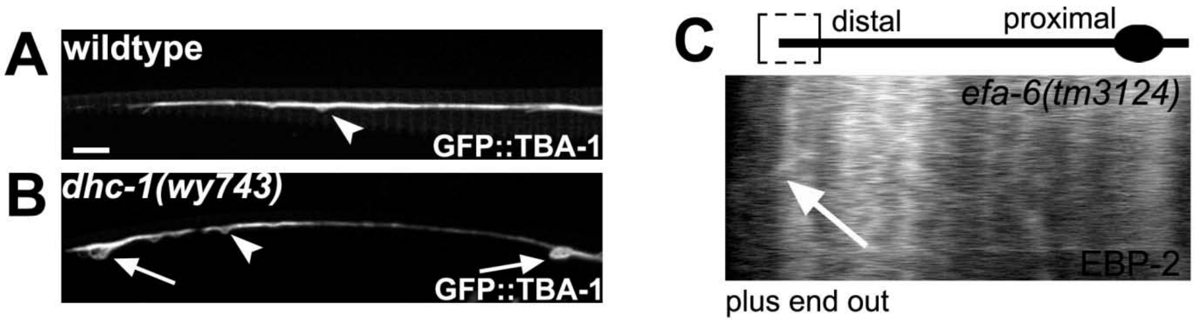

Supplementary Figure 7: *dhc-1* is required to prevent loop formation on a plus-end-out MT array

A and B show the tip of the DA1 axon. DA1, is the most anterior DA neuron, and its axon terminates near the anterior pharynx bulb .A. In a wt axon no loops are observed. A mild buckling of the axon is sometimes seen, mostly when the animal's head is bent (arrowhead). B. In *dhc-1(wy743)* mutants, there is a loop at the tip of the axon (arrow). An additional loop is seen more posteriorly, presumably at the tip of DA2 or DA3. Axonal MT loops in DA1 were mostly observed after one or two day of adulthood, unlike the DA9 dendrite loop, which can be detected as early as L3. The presence of loops at the tip of the axon, which is has uniformly polarized MTs pointing away from the cell body, indicates that *dhc-1* is required to prevent loop formation of plus-end-out MTs. C. An EBP-2::GFP comet is seen at the tip of the dendrite in *efa-6(tm3124)* mutants. *efa-6* mutants do not have MT polarity defects, but show a slight increase of MT dynamics in DA9.

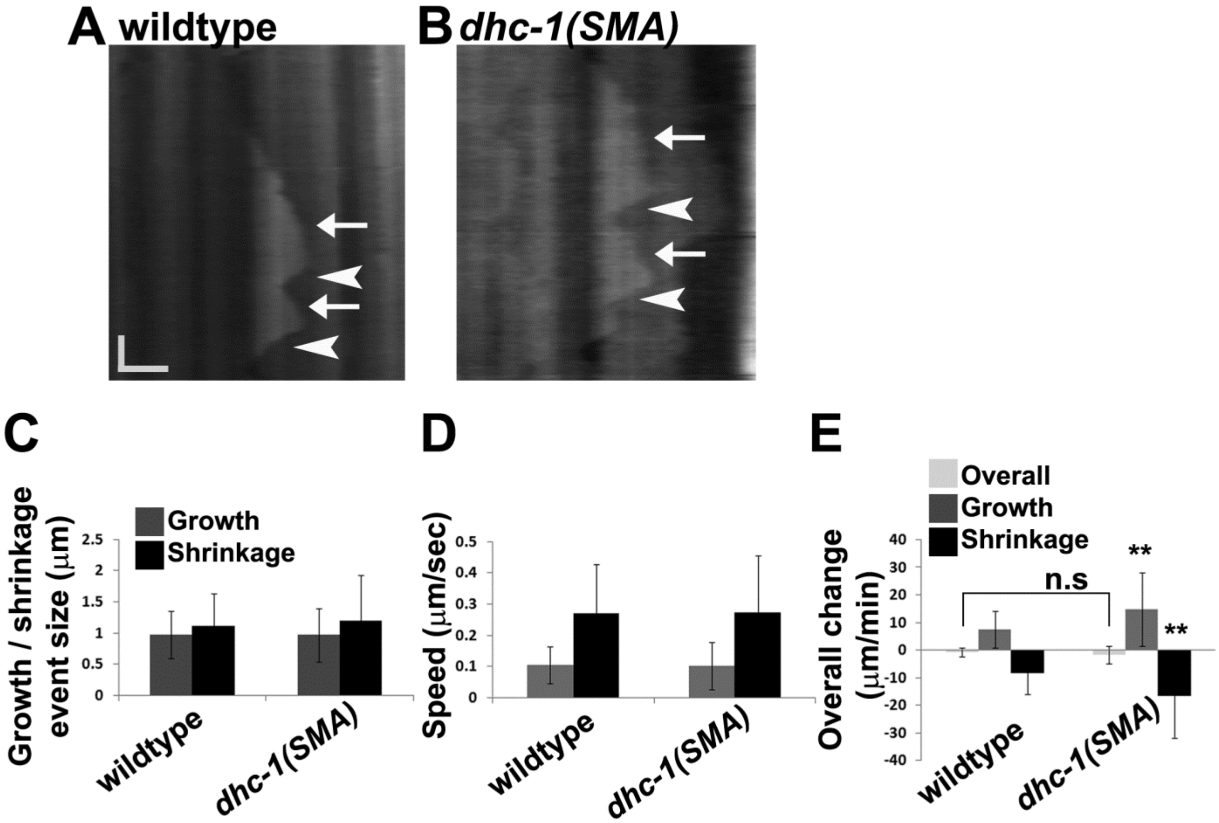

Supplementary Figure 8: DHC-1 acts to prevent excessive MT dynamics, not excessive growth

A and B. Kymographs form a GFP::TBA-1 movie of wildtype (A) and *dhc-1(SMA)* (B) dendrite. Scale bar is two microns/10secs. Unlike EBP-2::GFP, which reports only MT growth events, GFP::TBA-1 allows to follow both growth (arrows) and shrinkage (arrowheads). In both genotypes MT growth is almost always coupled to shrinkage. C-E. Quantifications of MT dynamic parameters. n=22-26 kymographs/genotype. **$p<0.01$. C. The average extent of individual growth/shrinkage events in *dhc-1(SMA)* is not different from wt, although it is more variable. D. MT growth speed and shrinkage speed is similar between wildtype and *dhc-1(SMA)*. E. Total MT growth is increased in *dhc-1(SMA)* dendrites compared to wt, but it is offset by increased shrinkage, such the total change is nearly zero, as in wildtype.

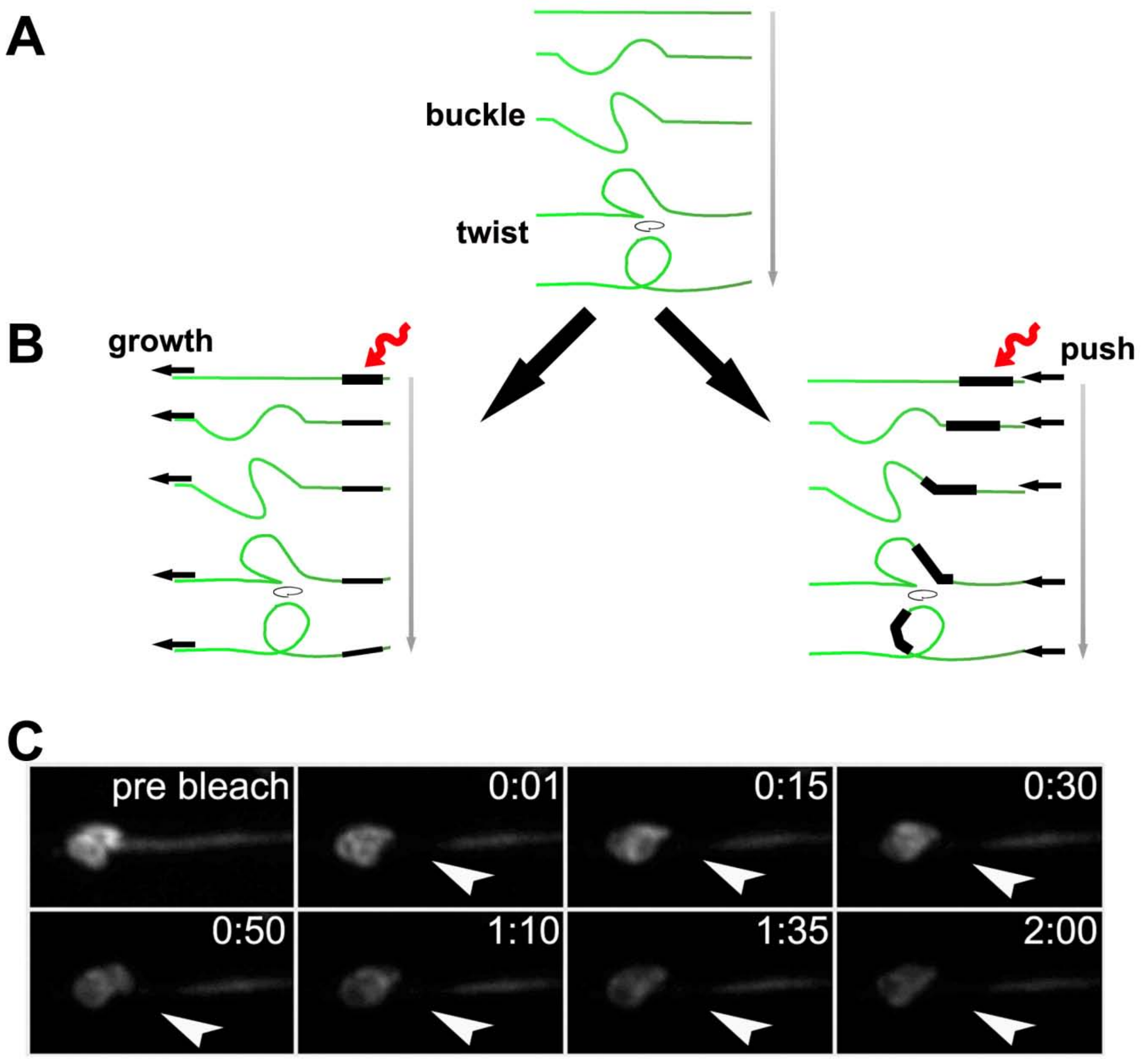

Supplementary Figure 9: MT looping occurs through buckling and twisting as MTs grow outward and does not involve MT movement into the loop

A. Scheme illustrating the sequence of events leading to loop formation as seen in GFP::TBA-1 and EMTB::GFP movies from *dhc-1* mutants. The buckling and twisting could result from a force that pushes MTs towards the distal tip, or from growth against a barrier at the tip. B. Scheme of expected results under each model. Right: If MTs are pushed towards the dendrite tip, then as the loop collapses and reforms, those MTs should move distally, and if they are marked by photobleaching, the photobleached mark should be observed in the loop. Left: If the buckling results from growth at the plus ends, then no MTs are expected to move into the loop region, even after several rounds of loops collapse and reformation. C. Images from a GFP::TBA-1 movie in *dhc-1(wy743)* mutants. The arrowhead indicates the position of the bleached region. Despite abundant dynamic behavior at the loop, the bleached region remains at the same place. Furthermore, no labeled MTs are observed crossing the bleached region. This result is consistent with MT loop formation being the result of growth at the tip rather than MT distal movement.

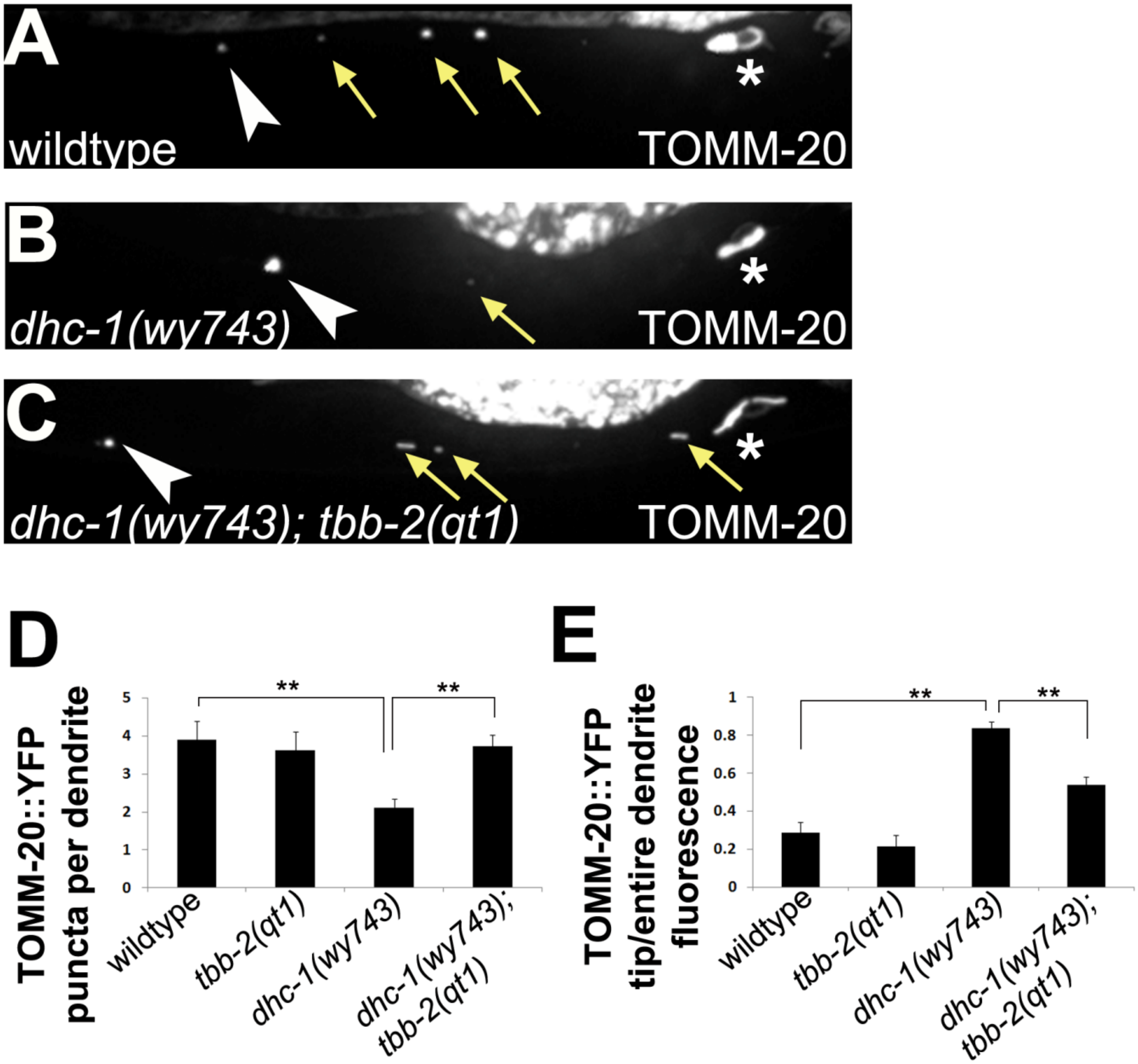

Supplementary Figure 10: MT stabilization reduces the mitochondrial tip accumulations in *dhc-1(wy743)* mutants

A. In wildtype animals the mitochondrial marker TOMM-20::YFP is evenly distributed along the dendrite. The distal-most punctum (white arrowhead) is usually not brighter than the other puncta (yellow arrows). * marks the cell body. The signal at the top of the image is coming from the gut, where the transgene is also expressed. B. In *dhc-1(wy743)* almost all the TOMM-20::YFP is concentrated at the tip of the dendrite. C. *dhc-1(wy743); tbb-2(qt1)* double mutants still show a bright TOMM-20::YFP punctum at the tip of the dendrite, but the signal is reduced compared to *dhc-1(wy743)* single mutants. This is accompanied by the re-appearance of additional TOMM-20::YFP puncta along the dendrite. *tbb-2(qt1)* is a mutation that enhances MT stability. D and E. Quantification of the number of TOMM-20::YFP puncta (D) and the fraction of TOMM-20::YFP fluorescence intensity that is localized to the tip of the dendrite (E). n=18-32, ** indicates $p<0.01$, multiple ANOVA.

Supplementary Table 1

Worm strains

Strain	Genotype
TV10640	*wySi265[Punc-4c::CRE]; wyEx4327[Pmig-13::LoxP::STOP::LoxP::SNG-1::GFP]*
TV13741	*dhc-1(wy743); wySi265[Punc-4c::CRE]; wyEx4327[Pmig-13::LoxP::STOP::LoxP::SNG-1::GFP]; wyEx1902[Pitr-1::mCherry]*
TV10884	*dhc-1(js319); wySi265[Punc-4c::CRE]; wyEx4327[Pmig-13::LoxP::STOP::LoxP::SNG-1::GFP]*
TV4913	*wyEx1902[Pitr-1::mCherry]*
TV12751	*dhc-1(wy743); wyEx1902[Pitr-1::mCherry]*
TV7359	*wyEx2709[Pitr-1::TOMM-20::YFP]*
TV13743	*dhc-1(wy743); wyEx2709[Pitr-1::TOMM-20::YFP]*
TV21716	*dhc-1(wy743); trak-1(tm1572); wyEx2709[Pitr-1::TOMM-20::YFP]*
TV13322	*trak-1(tm1572); wyEx2709[Pitr-1::TOMM-20::YFP]*
TV1229	*wyIs85[Pitr-1::GFP::RAB-3]*
TV15132	*dhc-1(wy743); wyIs85[Pitr-1::GFP::RAB-3]*
TV7049	*unc-104(e1265); wyIs85[Pitr-1::GFP::RAB-3]*
TV21715	*dhc-1(wy743); unc-104(e1265); wyIs85[Pitr-1::GFP::RAB-3]*
TV8456	*dhc-1(js319); wyIs85[Pitr-1::GFP::RAB-3]*
TV9778	*wyEx2927[Punc-129::GFP::RAB-3; Pmig-13::mCherry::RAB-3]*
TV16693	*dhc-1(js319); wyEx2927[Punc-129::GFP::RAB-3; Pmig-13::mCherry::RAB-3]*
TV15986	*dhc-1(wy743); wyEx2927[Punc-129::GFP::RAB-3; Pmig-13::mCherry::RAB-3]*
TV21592	*unc-104(e1265); wyEx2927[Punc-129::GFP::rab-3, Pmig-13::mCherry::rab-3]*
TV21714	*dhc-1(wy743); unc-104(e1265); wyEx2927[Punc-129::GFP::rab-3, Pmig-13::mCherry::rab-3]*
TV6804	*dhc-1(or195); wyIs85[Pitr-1::GFP::RAB-3]*
TV21713	*dhc-1(or352); wyIs85*
TV20726	*dhc-1(SMA); wyIs85[Pitr-1::GFP::RAB-3]*
TV21712	*dhc-1(wy743); wyEx3194[genomic dhc-1 fragment]*
TV21711	*dhc-1(LOA); wyIs85*
TV21617	*dhc-1(LOA); wySi265[Punc-4c::CRE]; wyIs463[Pmig-13::LoxP::STOP::LoxP::GFP::TBA-1]; wyIs363[Pmig-13::LoxP::STOP::LoxP::tdtomato::RAB-3]*
TV16538	*wyIs85[Pitr-1::GFP::RAB-3]; wyEx6641[dhc-1(wy743) genomic fragment]*
TV21709	*wyIs85[Pitr-1::GFP::RAB-3]; wyEx6689[dhc-1(js319) genomic fragment]*
TV21710	*wyIs85[Pitr-1::GFP::RAB-3]; wyEx6688[dhc-1 genomic fragment]*
TV13204	*trak-1(tm1572); wyIs85[Pitr-1::GFP::RAB-3]*
TV21708	*dhc-1(wy743), trak-1(tm1572); wyIs85[Pitr-1::GFP::RAB-3]*
TV9724	*wyEx3892[Pitr-1::GFP::DHC-1]*
TV12750	*dhc-1(wy743); wyEx3892[Pitr-1::GFP::DHC-1]*
TV9430	*wyEx3713[Pitr-1::dlc-1::GFP]*
TV19135	*dhc-1(wy743); wyEx3713[Pitr-1::dlc-1::GFP]*
TV20976	*wyEx3713[Pitr-1::dlc-1::GFP]; wyEx1902[Pitr-1::mCherry]*

TV13169	*wyEx3892[Pitr-1::GFP::DHC-1]; wyEx1902[Pitr-1::mCherry]*
TV21707	*dhc-1(wy743); wyEx3892[Pitr-1::GFP::DHC-1]; wyEx1902[Pitr-1::mCherry]*
TV21706	*dhc-1(wy743); wyEx3713[Pitr-1::dlc-1::GFP]; wyEx1902[Pitr-1::mCherry]*
TV10888	*dhc-1(js319); wySi265[Punc-4c::CRE]; wyEx4327[Pmig-13::LoxP::STOP::LoxP::SNG-1::GFP]*
TV14328	*dhc-1(wy743); wyEx2873[Pitr-1::unc-104::GFP]*
TV9500	*wyIs674[Pitr-1::KLP-16::YFP]*
TV12852	*dhc-1(wy743); wyIs674[Pitr-1::KLP-16::YFP]*
TV14692	*dhc-1(or195); wySi265[Punc-4c::CRE]; wyIs674[Pitr-1::KLP-16::YFP]; wyIs363[Pmig-13::LoxP::STOP::LoxP::tdtomato::RAB-3]*
TV13788	*dhc-1(or352); wyEx5296[Pitr-1::3XGFP::EMTB]*
TV21705	*dhc-1(LOA); wyIs674[Pitr-1::KLP-16::YFP]*
TV13205	*trak-1(tm1572); wyIs674[Pitr-1::KLP-16::YFP]*
TV21704	*dhc-1, trak-1(tm1572); wyIs674[Pitr-1::KLP-16::YFP]*
TV21082	*dhc-1(SMA); wyIs674[Pitr-1::KLP-16::YFP]*
TV8594	*dhc-1(js319); wyEx3128[Pitr-1::KLP-16::YFP]*
TV14653	*dhc-1(wy743)/hT2; wyEx3892[Pitr-1::GFP::DHC-1]; wyEx3128[Pitr-1::KLP-16::YFP]*
TV16674	*wyIs674[Pitr-1::KLP-16::YFP]; wyEx6688[dhc-1 genomic fragment]*
TV16675	*wyIs674[Pitr-1::KLP-16::YFP]; wyEx6689[dhc-1(js319) genomic fragment]*
TV16538	*wyIs674[Pitr-1::KLP-16::YFP]; wyEx6641[dhc-1(wy743) genomic fragment]*
TV17136	*dhc-1(wy743); wyIs324/+; wyEx6864*
TV14626	*wySi265[Punc-4c::CRE]; wyIs463[Pmig-13::LoxP::STOP::LoxP::GFP::TBA-1]*
TV17203	*dhc-1(wy743); wySi265[Punc-4c::CRE]; wyIs463[Pmig-13::LoxP::STOP::LoxP::GFP::TBA-1]*
TV19492	*wySi265[Punc-4c::CRE]; wyIs463[Pmig-13::LoxP::STOP::LoxP::GFP::TBA-1], wyIs720[Pmig-13::LoxP::STOP::tagRFP::PTRN-1]*
TV20003	*dhc-1(wy743); wySi265[Punc-4c::CRE]; wyIs463[Pmig-13::LoxP::STOP::LoxP::GFP::TBA-1], wyIs720[Pmig-13::LoxP::STOP::tagRFP::PTRN-1]*
TV20944	*dhc-1(SMA); wySi265[Punc-4c::CRE]; wyIs463[Pmig-13::LoxP::STOP::LoxP::GFP::TBA-1], wyIs720[Pmig-13::LoxP::STOP::tagRFP::PTRN-1]*
TV21018	*dhc-1(wy743); wySi265[Punc-4c::CRE]; tbb-2(qt1); wyIs463[Pmig-13::LoxP::STOP::LoxP::GFP::TBA-1], wyIs720[Pmig-13::LoxP::STOP::tagRFP::PTRN-1]*
TV21699	*dhc-1(js319); wyIs85[Pitr-1::GFP::RAB-3]; wyEx8604[Pmig-13::myrGFP::DHC-1]*
TV21697	*dhc-1(js319); wyIs85[Pitr-1::GFP::RAB-3]; wyEx3892[Pitr-1::GFP::DHC-1]*
TV21700	*dhc-1(wy743); wyIs85[Pitr-1::GFP::RAB-3]; wyEx8604[Pmig-13::myrGFP::DHC-1]*
TV21698	*dhc-1(wy743); wyIs85[Pitr-1::GFP::RAB-3]; wyEx3892[Pitr-1::GFP::DHC-1]*
TV21701	*wyEx8604[Pmig-13::myrGFP::DHC-1]*
TV20961	*dhc-1(SMA); wySi265[Punc-4c::CRE]; wyIs463[Pmig-13::LoxP::STOP::LoxP::GFP::TBA-1]; wyIs363[Pmig-13::LoxP::STOP::LoxP::tdtomato::RAB-3]; wyEx8604[Pmig-13::myrGFP::DHC-1]*

TV20725	*dhc-1(SMA); wySi265[Punc-4c::CRE]; wyIs463[Pmig-13::LoxP::STOP::LoxP::GFP::TBA-1]; wyIs363[Pmig-13::LoxP::STOP::LoxP::tdtomato::RAB-3]*
TV21703	*dhc-1(743); wySi265[Punc-4c::CRE]; wyIs463[Pmig-13::LoxP::STOP::LoxP::GFP::TBA-1]; wyIs363[Pmig-13::LoxP::STOP::LoxP::tdtomato::RAB-3]; wyEx8604[Pmig-13::myrGFP::DHC-1]*
TV17356	*wySi265[Punc-4c::CRE]; wyIs463[Pmig-13::LoxP::STOP::LoxP::GFP::TBA-1]; wyIs363[Pmig-13::LoxP::STOP::LoxP::tdtomato::RAB-3]*
TV20757	*dhc-1(SMA); wySi265[Punc-4c::CRE]; wyIs463[Pmig-13::LoxP::STOP::LoxP::GFP::TBA-1]; jnk-1(gk7); wyIs363[Pmig-13::LoxP::STOP::LoxP::tdtomato::RAB-3]*
TV21702	*dhc-1(743); wySi265[Punc-4c::CRE]; wyIs463[Pmig-13::LoxP::STOP::LoxP::GFP::TBA-1]; wyIs363[Pmig-13::LoxP::STOP::LoxP::tdtomato::RAB-3]*
TV13069	*wyEx5296[Pitr-1::3XGFP::EMTB]*
TV21694	*unc-116(e2310); wyEx5296[Pitr-1::3XGFP::EMTB]*
TV14548	*dhc-1(wy743/+); unc-116(e2310); wyEx5296[Pitr-1::3XGFP::EMTB]*
TV12240	*wySi265[Punc-4c::CRE]; wyIs364[Pmig-13::LoxP::STOP::LoxP::EBP-2::GFP]*
TV21692	*wySi265[Punc-4c::CRE]; wyEx3892[Pitr-1::GFP::DHC-1]; wyEx7468[Pmig-13::LoxP::STOP::tagRFP::PTRN-1]*
TV10622	*unc-116(e2310); wyEx3128[Pitr-1::KLP-16::YFP]*
TV17239	*dhc-1(wy743/+); unc-116(e2310); wyEx3128[Pitr-1::KLP-16::YFP]*
TV20975	*unc-116(e2310); wyEx3713[Pitr-1::dlc-1::GFP]; wyEx1902[Pitr-1::mCherry]*
TV13784	*dhc-1(wy743); wySi265[Punc-4c::CRE]; wyIs364[Pmig-13::LoxP::STOP::LoxP::EBP-2::GFP]*
TV14674	*wySi265[Punc-4c::CRE]; efa-6(tm3124); wyIs364[Pmig-13::LoxP::STOP::LoxP::EBP-2::GFP]*
TV15080	*dhc-1(wy743); wySi265[Punc-4c::CRE]; tbb-2(qt1); wyIs364[Pmig-13::LoxP::STOP::LoxP::EBP-2::GFP]*
TV21693	*tbb-2(qt1); wySi265[Punc-4c::CRE]; wyIs364[Pmig-13::LoxP::STOP::LoxP::EBP-2::GFP]*
TV15682	*tbb-2(qt1); wyEx3128[Pitr-1::KLP-16::YFP]*
TV15082	*dhc-1(wy743); tbb-2(qt1); wyEx3128[Pitr-1::KLP-16::YFP]*
TV15686	*tbb-2(qt1); wyIs85[Pitr-1::GFP::RAB-3]*
TV15130	*dhc-1(wy743); tbb-2(qt1); wyIs85[Pitr-1::GFP::RAB-3]*
TV21696	*tbb-2(qt1); wyEx2709[Pitr-1::TOMM-20::YFP]*
TV21695	*dhc-1(wy743); tbb-2(qt1); wyEx2709[Pitr-1::TOMM-20::YFP]*
TV13272	*dhc-1(wy743); wyEx5296[Pitr-1::3XGFP::EMTB]*
